# Hybrid physics-machine learning models for predicting rate of penetration in the Halahatang oil field, Tarim Basin

**DOI:** 10.1038/s41598-024-56640-y

**Published:** 2024-03-12

**Authors:** Shengjie Jiao, Wei Li, Zhuolun Li, Jingming Gai, Linhao Zou, Yinao Su

**Affiliations:** 1 School of Petroleum Engineering, Northeast Petroleum University, Daqing, China; 2 National Engineering Research Center of Oil & Gas Drilling and Completion Technology, Beijing, China

**Keywords:** Prediction of the rate of penetration, Physical model, Machine learning, Hybrid model, Engineering, Physics

## Abstract

Rate of penetration (ROP) is a key factor in drilling optimization, cost reduction and drilling cycle shortening. Due to the systematicity, complexity and uncertainty of drilling operations, however, it has always been a problem to establish a highly accurate and interpretable ROP prediction model to guide and optimize drilling operations. To solve this problem in the Tarim Basin, this study proposes four categories of hybrid physics-machine learning (ML) methods for modeling. One of which is residual modeling, in which an ML model learns to predict errors or residuals, via a physical model; the second is integrated coupling, in which the output of the physical model is used as an input to the ML model; the third is simple average, in which predictions from both the physical model and the ML model are combined; and the last is bootstrap aggregating (bagging), which follows the idea of ensemble learning to combine different physical models’ advantages. A total of 5655 real data points from the Halahatang oil field were used to test the performance of the various models. The results showed that the residual modeling model, with an R^2^ of 0.9936, had the best performance, followed by the simple average model and bagging with R^2^ values of 0.9394 and 0.5998, respectively. From the view of prediction accuracy, and model interpretability, the hybrid physics-ML model with residual modeling is the optimal method for ROP prediction.

## Introduction

Drilling is a costly, high-impact, mission-critical operation, and the ROP is a key indicator of drilling efficiency. A higher ROP indicates faster drilling, increased rig productivity, and better rig performance^1^. To obtain a higher ROP, it is necessary to construct a model that can evaluate how drilling variables affect ROP, and a large number of scholars and researchers have comprehensively analyzed actual drilling operations and found many factors that affect ROP, such as drill bits, weight on bit (WOB), revolutions per minute (RPM). Combined with laboratory experiments and theoretical analysis, under certain assumptions, a variety of ROP models suitable for different working conditions have been proposed to describe and predict ROP. These models can be divided into two types: classical physical models and ML models. In general, obtaining an ROP model can be seen as a regression problem. The basic research method is to use the drilled drilling curve to fit a model, and the parameters to be fitted include: empirical coefficients from the physical model, and internal hyperparameters from the ML model^2^.

Classical physical models are rigorous mathematical equations established by theoretical analysis or experimentation between drilling variables. Some classical physical models that are often mentioned in research or widely used in actual drilling including Bingham^3^, Eckel^4^, Young^5^, Bourgoyne and Young^6^, Soares^7^ and other classic physical models. These physical models can be broadly understood as any knowledge that expresses the effective relationship between the properties or elements of drilling objects^8^, including physical knowledge, geometric constraints, stratigraphic laws, etc. Physical models follow objective laws and establish explicit associations between inputs and outputs to help people recognize and understand the physical world in which they live^9^. The physical model can clearly describe the internal characteristics of the system, and its outstanding advantages include its rigorous theory, (relatively) stable model, and interpretable results. However, physical models also have insurmountable shortcomings:

1. Limitation of the understanding of drilling downhole physics. A drilling system is a complex system^10^ with mixed elements, multiscale coupling and multiple process intertwined; however, accurately depicting all drilling processes is still difficult to in the existing physical model, and some physical processes are still unknown. For example, in the actual drilling process, the influence of drilling fluid on ROP is complex, and the influence of drilling fluid displacement, viscosity and density on ROP is not clearly understood, resulting in the assumption and simplification of physical model modeling and thereby triggering uncertainty in ROP prediction.
2. Underdetermined system problems. Even if most physicals in drilling processes are clear, some parameter inversions are often underdetermined systems; that is, the number of observation equations is less than the number of unknown parameters, resulting in unstable effects of the drilling speed model. For example, in the actual drilling process, the heterogeneity of rocks and the wear of drill bits cannot be directly observed, and simple assumptions and some deductions will affect the effectiveness of the physical model.
3. Insufficient accuracy. Despite making a lot of efforts (theoretical and experimental), modeling the ROP as a mathematical function of some variables is not trivial because this is a highly non-linear problem^2^. The systematic, complex and uncertain drilling conditions of the downhole drilling process and the limitations of traditional ROP modeling result in a lack of sufficiently high accuracy.

Due to the limitations of physical models and the development of ML algorithms and the improvement in computer computing power, data-driven ML methods have been widely applied to ROP prediction and have achieved good results. One of the earliest applications of ML methods for ROP prediction dates back to 1997. Bilgesu creatively designed a one-hidden layer feed-forward back-propagation network to predict ROP and demonstrated the applicability of this neural network approach^11^. Mendes^12^ also presented a methodology based on a neural network model for ROP and a neuro-genetic adaptive controller to address the problem that relationships between operational variables affecting ROP are complex and not easily modeled. In addition, with the boom in ML algorithms approximately 2010, more and more ML methods are being used for ROP prediction, including Moran^13^, Arabjamaloei^14^, Esmaeili^15^, Ning^16^, Zare^17^, Bodaghi^18^, Hegde^19^, Mantha^20^, Hegde^21^, Anemangely^22^, Soares^7^, Sabah^23^, Felipe^2^, Korhan^24^, Li^25^, Mohammad^26^, Gan^27^, Hazbeh^28^, Salaheldin^29^, Zhang^30^, Ren^31^, Zhang^32^, Brenjkar^33^, Riazi^34^, Song^35^, Wang^36^, Mohammad^37^, Kaveh^38^ and so on. Judging from the increasing number of articles published each year in recent years on the use of machine learning for ROP prediction, it can be amply demonstrated that ML methods are well suited for application in the field of ROP prediction.

The ML methods used in the more than thirty articles mentioned above are all different, and to provide a clearer picture of the specifics of ML used for ROP prediction, the above articles are categorized according to the five ML algorithm types: artificial neural network (ANN), support vector machines (SVM), random forests (RF), hybrid model and deep learning (DL) methods. For each method, an additional information is given in the field of detail, and in the “Detail” column, the models used for comparison in the articles are in parentheses, as shown in Table 1.

**Table 1:** Classification of ML methods employed in ROP prediction.

Algorithm	Detail	References
ANN	Three-layer feed-forward back propagation	Bilgesu^11^, Mendes^12^, Moran^13^, Arabjamaloei^14^
	Multi-layer perceptron (MLP) networks trained with a back-propagation algorithm (BP)	Esmaeili^15^, Ning^16^, Zare^17^, Anemangely^22^
	MLP with particle swarm optimization algorithm (PSO) (MLP, a radial basis function (RBF) ANN, SVM)	Sabah^23^
	ANN with an improved genetic algorithm (IGA)	Li^25^
	MLP with Firefly algorithm (FF), Gravitational search algorithm (GSA), Artificial bee colony algorithm (ABC), Independent component analysis (ICA)	Hazbeh^28^
	ANN with 1 hidden layer, 20 neurons, fitnet as a network function, trainbr as a training function, tansig as a transfer function (adaptive neuro-fuzzy inference system (ANFIS), SVM)	Salaheldin^29^
	ANN with extreme learning machine (ELM)	Gan^27^
	combining an attention-based Gated Recurrent Unit network and fully connected neural networks	Zhang^32^
	multilayer perceptron neural network (MLPNN), radial basis function neural network (RBFNN) (adaptive neuro-fuzzy inference system (ANFIS), and support vector regression (SVR))	Brenjkar^33^
	MLP with Bayesian Regularization Algorithm (BRA) (Radial Basis Function, Decision Tree (DT), Least Square Vector Machine (LSSVM))	Mohsen^34^
SVM	Support vector regression with the genetic algorithm (GA) and the cuckoo search algorithm (CS)	Bodaghi^18^
	least-squares support-vector machines (LSSVM) with cuckoo optimization algorithm (COA), particle swarm optimization (PSO), and genetic algorithms (GA) (SVR-COA, MLP-COA, linear multivariate regression (LMR), and nonlinear multivariate regression (NLMR))	Mohammad^26^
	ε-insensitive SVR and V-SVR	Korhan^24^
RF	RF (Trees, Bagging)	Hegde^19^
	RF (ANN, SVM, KNN (k-nearest neighbor), decision trees (DT))	Mantha^20^
	RF (SVM, BP, KNN, RBF Network)	Zhang^30^
	RF (SVM, ANN)	Soares^7^, Song^35^
	RF (MLP)	Kaveh^38^
Hybrid models	Traditional models with RF, ANN, Linear regression	Hegde^21^
	Traditional models with RF, ELM, BP, SVM	Ren^31^
DL	long short- term memory (LSTM) neural network, SVR, BP, deep belief neural network (DBN), convolutional neural network (CNN)	Wang^36^
	Generative Adversarial Network (GAN), MLP, CNN	Mohammad^37^

Table 1 shows a history of the application of ML models in predicting ROP, and the more commonly used methods include ANN, SVM, RF and hybrid models. Moreover, recent years have also seen the emergence of approaches involving hybrid models and DL for ROP prediction. For example, in 2016, B. Mantha et al. realized the use of Step-Wise regression, neural networks (NN, KNN), support vector regression (SVR), categorical regression trees (CART) and other models for prediction^20^. Ensemble methods such as RF and Boosting help improve accuracy and reduce errors.

In 2022, based on data-driven thinking, Zhang proposed solving the real-time optimization problem of ROP by combining attention-based Gated Recurrent Unit networks and fully connected neural networks, which are accurate and robust and can make predictions after training on the first few data streams^32^. Compared with the traditional data-driven model, the proposed model shows great superiority due to its subnetwork structure, gated loop unit network and attention physical.

In 2022, Gan used a mobile window strategy, extreme learning machines, and tenfold cross-validation to build an ROP model^27^. Alternately, two steps (modeling and forecasting) are performed within a moving drilling depth window to more accurately predict the ROP.

In 2023, Zhang used RF, ANN and SVM combined with real-time workflow to predict drilling speed in real time^35^ and optimized drilling parameters through the NSG- III algorithm through an objective function for ROP and MSE to obtain a better real-time prediction effect during drilling.

Considering the above for the application of ML methods in ROP prediction, ML models simulate the “inductive” and “speculative” processes of humans through “training” and “prediction” respectively, to model and solve typical problems. Unlike the explicit expression of mechanistic models, ML models establish implicit associations between different variables through training on data; this is often a typical “black box” model. One of the key advantages of ML models is that when physical activity is unknown, they can skip the understanding of the physical process and go directly to data-driven modeling, especially when the training data are sufficient, and high modeling accuracy can often be obtained. In addition, although ML is time-consuming in the training stage, it generally has high computational efficiency in the test application stage, which has become one of its important advantages. Despite this, ML still has some limitations, especially in the drilling process of complex processes, which often have the following problems:

1. Insufficient generalization. The lack of sufficient training samples is the most common problem of ML in drilling applications, and the use of limited samples to learn complex drilling processes is prone to overfitting, even if the training samples show high modeling accuracy, the test application accuracy will be greatly reduced. In particular, when the actual numerical range, variable relationship, etc. are not covered by the training sample, the prediction result is more likely to be extremely biased, that is, the typical generalization ability is insufficient.
2. Insufficient migration. Regionality is the essential characteristic of rocks in drilling, and the differences between different regions manifest not only as differences in different geological elements but also as differences in the relationships between various elements. As a result, it is often difficult for ML models trained in one region to migrate to other regions for application. Second, the Earth’s surface elements and their interrelationships are undergoing constant change, and human activities make them more drastic; therefore, models of different time spans in the same region are often difficult to apply. In addition, insufficient scale migration is another dilemma in drilling applications.
3. Insufficient interpretability. The goal of scientific research is not only to develop a usable model, but also to discover the intrinsic causal relationships and driving patterns between different variables, and use them to explain theories and hypotheses, thereby contributing to the advancement of scientific knowledge^9^. One of the outstanding problems of ML is the lack of interpretability; although it can also obtain relatively high accuracy under specific conditions, lacks the ability to explain the internal physical process.

According to the result of the analysis of the application of ROP prediction models in different articles, the physical models and the ML models have different characteristics for ROP prediction and no single ROP model is suitable for all drilling site conditions. More importantly, most of the above studies have updated physical models or fused ML models to improve accuracy, but few studies have combined physical models and ML models to improve both accuracy and interpretability. On the one hand, the construction methods of the hybrid models are various, and it is uncertain which kind of construction method has more accurate prediction results. On the other hand, there are kinds of physical and ML models in the hybrid models, and it is also unknown which combination of physical and ML models is the best. Therefore, it is hard to satisfy the high-accuracy and great interpretability requirement for ROP prediction by the previous method, and it is necessary to deepen the studies on hybrid models of ROP prediction.

To construct an ROP prediction model for the Halahatang oil field that achieves high prediction accuracy and maintains a certain degree of interpretability, this paper combines the advantages of different physical models and ML models to propose four novel hybrid physics-ML models because there is a growing consensus that solutions to complex science and engineering problems require novel methodologies that are able to integrate traditional physics-based modeling approaches with state-of-the art ML techniques. This paper starts from the operation parameters that can be conveniently controlled by a field driller: WOB, RPM and Q. On the basis of several classical physical models, combined with commonly used ML methods for data training and experiments, the two parameters are combined to construct an ROP prediction model. There are four novel kinds of hybrid modeling approaches designed to reach this goal. The results obtained from this study can be applied in drilling parameter optimization and ROP management of drilling wells to add technical and economic benefits in the future^31^.

## Methodology

### Procedure of the hybrid physics-ML ROP modeling

**Figure 1:**
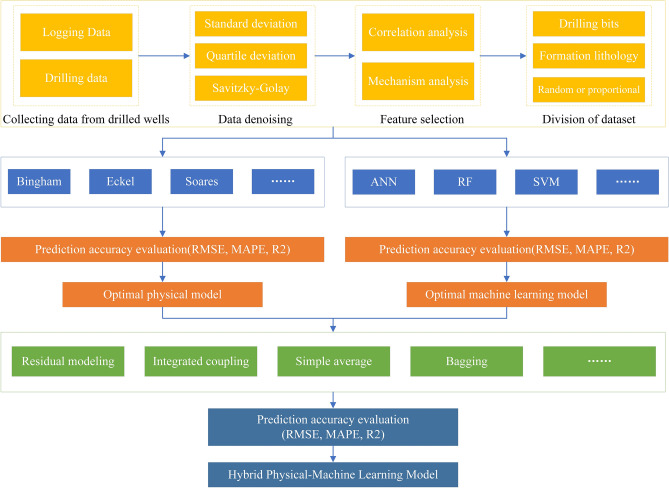
Procedure of the hybrid ROP modeling.

In general, no ROP prediction model can be adapted for all drilling fields. From the previous demonstration, physical models and ML models have different advantages; perhaps the hybrid physics-ML ROP prediction model which combines physical models and ML models can have better accuracy and interpretability. The hybrid ROP prediction model may be more suitable as long as it is rebuilt before it is used in new drilling fields via the hybrid ROP modeling procedure. The procedure is shown in Fig. 1.

On the top of Fig. 1, four stages, which include collecting data from drilled wells, data denoising, feature selection, division of dataset are used for data processing before modeling. In the middle of Fig. 1, the goal of this stage is to select suitable physical and ML models from among the different models through three model performance evaluation metrics. On the bottom of Fig. 1, this study puts forward four novel approaches for hybrid physics-ML modeling, which include residual modeling, integrated coupling, simple average and bagging, maybe in the future, there will be more approaches for hybrid modeling. At the end of the procedure, a suitable model for this field will be selected by comparing the RMSE, MAPE and R^2^. However, perhaps the best hybrid model is not unitary because of the characteristics of different regions despite having the same modeling procedure.

### Tarim Basin (Xinjiang) dataset

**Figure 2:**
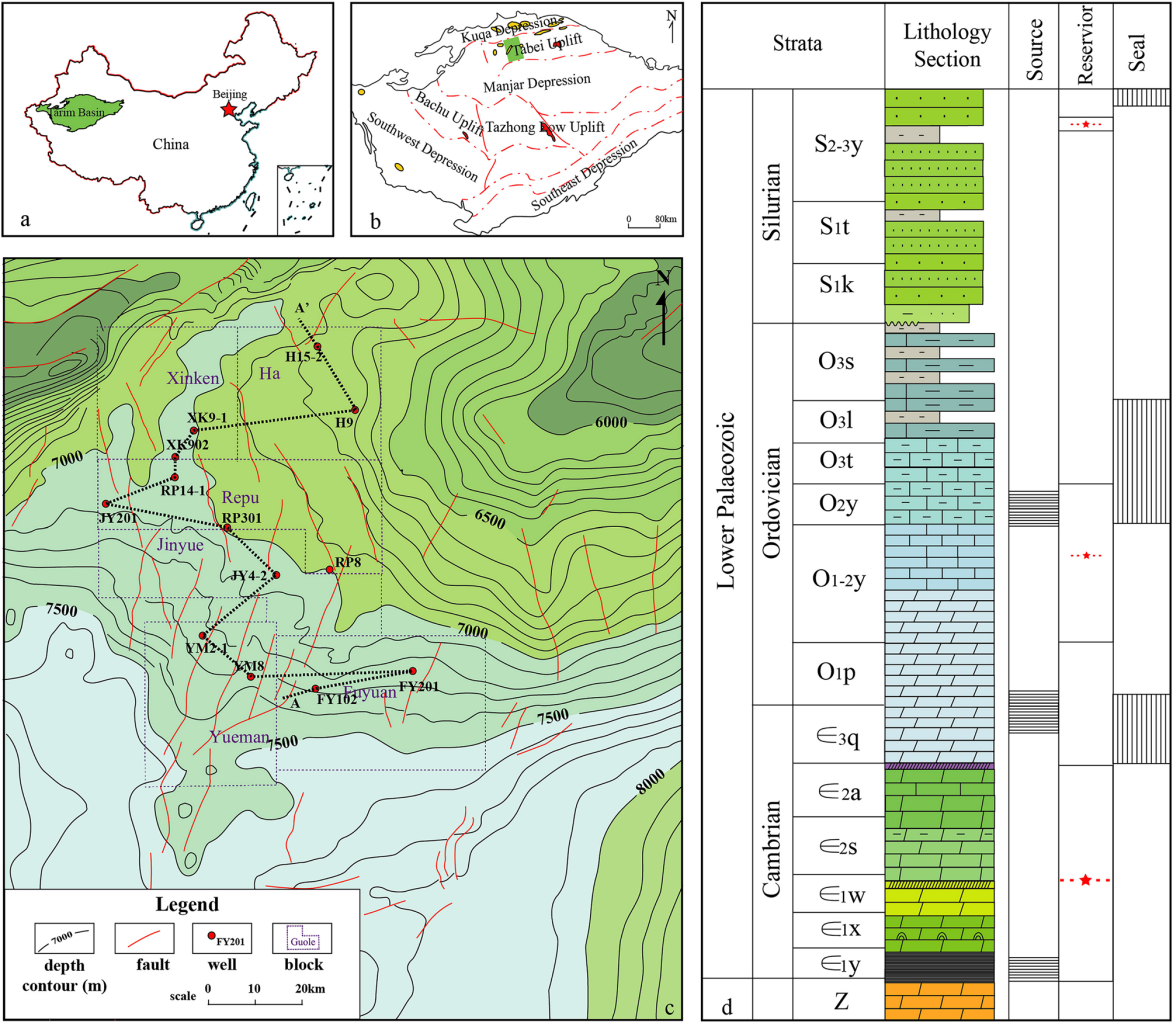
Generalized geologic setting of the Halahatang region^39^. (**a**) Location of the Tarim Basin, China; (**b**) location of the study area in the northern Tarim Basin; (**c**) structural map of the Halahatang oil field; (**d**) and comprehensive stratigraphic column for the northern Tarim Basin.

In this study, comprehensive logging data from an ultra-deep well in the Halahatang oil field of the Tarim Basin in southern Xinjiang were used. The Halahatang area is the main oil-bearing zone in the Tarim Basin, and the Ordovician-rich carbonate-rich oil and gas resources in this area, which are deep unconventional oil and gas resources, are buried at a depth of more than 7000 m (Fig. 2a–c). Due to the drilling depth generally above 7000 m, the lithology is carbonate, and the drilling period is long (Fig. 2d).

The basic data of this well is shown in Table 2, the measured data relevant to ROP are composed of formation parameters including dc exponent (DC), normal dc exponent (DCN), formation pressure gradient (${g}_{p}$), formation fracture pressure gradient (${G}_{f}$), Poisson’s ratio ($\mu$), drilling parameters containing WOB, RPM, drill fluid flow rate (Q), torque (TORQUE), drilling time (DT), hook load (HL), stand pipe pressure (SPP), well trajectory parameters including measured depth (MD), true vertical depth (TVD), downhole parameters consisting of drill-bit diameter (${d}_{b}$), drill-bit footage (${d}_{f}$), and drilling fluid parameters containing drilling fluid equivalent density (ECD).

**Table 2:** Dataset basics.

Name	Abbreviation	Unit	Average	Maximum	Minimum	Median	Variance
Measured depth	MD	m	4334.5	7162.0	1507.0	4334.50	2,665,868
Hook load	HL	kN	1550	2450	635	1589	206,949
Bit revolution per minute	RPM	rev/min	74	161	1.0	71	189
Drilling time	DT	Min/m	7	113	1.9	5	63
Rate of penetration	ROP	m/h	20.9	120	1.0	12	474
Weight on bit	WOB	kN	51.6	178	1.0	41	1028
Torque	TORQUE	kN·m	4.7	9.0	0.0	5	2.1
Stand pipe pressure	SPP	MPa	18.0	22	8.0	19	5.6
Drill fluid flow rate	Q	l/min	2324.9	3685.4	1086.3	1903.1	405,329
DC exponent	DC	/	0.9	2.1	0.4	1.7	0.1
Drilling fluid equivalent density	ECD	g/cm^3^	1.2	1.3	1.1	1.3	0.0
Poisson’s ratio	$\mu$	/	0.7	2.3	0.1	0.6	0.1
Normal DC	DCN	/	1.0	1.4	0.5	0.0	0.9
Formation pressure gradient	${g}_{p}$	g/cm^3^	2.1	2.4	1.7	2.1	0.0
Formation fracture pressure gradient	${G}_{f}$	g/cm^3^	1.1	3.4	0.6	1.0	0.0

Ten PDC drill bits were used in the well, two sizes, 406.4 mm and 241.3 mm. The entire well spans 10 different strata including the Quaternary, Neogene, Paleogene, Cretaceous, Jurassic, Triassic, Carboniferous, Devonian, Silurian and Ordovician strata. Due to the serious lack of data for the first drill bit and the tenth drill bit in the logging data, only the second to eighth drill bits were selected for experimentation, and the different drill bits drilled into the well section are shown in Table 3:

**Table 3:** Different drill bits are drilled into the well section.

Order	Bit model	Bit diameter (mm)	Start depth (m)	End depth (m)	Footage (m)	Drill time (h)
1	16" HT2565B	406.4	0.0	1506.4	1506.4	77:30
2	9 1/2" TS1952	241.3	1506.4	3244.0	1736.6	83:15
3	9 1/2" FX55DI	241.3	3244.0	3660.0	416.0	62:00
4	9 1/2" TS1952	241.3	3660.0	5023.0	1363.0	161:00
5	9 1/2" SF55H3	241.3	5023.0	5404.0	381.0	125:00
6	9 1/2" U513M	241.3	5404.0	6672.0	1268.0	210:00
7	9 1/2"FX55SX	241.3	6672.0	6970.0	298.0	61:30
8	9 1/2" HJ517G	241.3	6970.0	7001.0	31.0	29:00
9	9 1/2" M1665	241.3	7001.0	7162.0	161.0	79:30
10	9 1/2" SF55H3	241.3	7162.0	7176.0	14.0	19:00

Post drilling data analysis and preprocessing from the preliminary description of the data in Table 2 reveal that due to sensor errors or measurement errors, several zero value points or mutation values need to be processed, to improve the data quality and lay a good foundation for subsequent model fitting.

### Data denoising

According to the basic statistics of the dataset in Table 2, the data quality of individual variables is poor, such as the variance in Q is large, there are obvious abnormal peaks and valleys in the distribution of Q, so data noise reduction is needed to improve the quality of data. Different data denoising methods may be suitable for different datasets and three data denoising methods are selected to improve the quality of data including standard deviation, quartile deviation and Savitzky–Golay (SG).

The preprocessing of data was divided into four situations: one was the raw data (as a control group), one was to determine the abnormal data according to a distance of 3 times the standard deviation, one was to preprocess the data according to 1.5 times the quartile difference, and the other was to smooth the data according to the SG algorithm. The SG smoothing filter^40^ is used to remove some of the noise in the original dataset. This method applies a polynomial function to reduce noise in data variables, replacing values identified as noise in the data records with values generated by the SG function^23^. Based on the identified least squares error, the nth order polynomial function is derived from the selected drill encounter stratigraphic points.

The number of points chosen should be odd and greater than the order of the derived polynomial function. If a higher polynomial order is applied, or if the number of data records fitted within that interval decreases, the derived polynomial function preserves the data trend of the variable. However, a decrease in the order of the polynomials or an increase in the number of data records used to define a particular interval can disrupt the data trend of the variable, resulting in excessive smoothing of the data.

**Figure 3:**
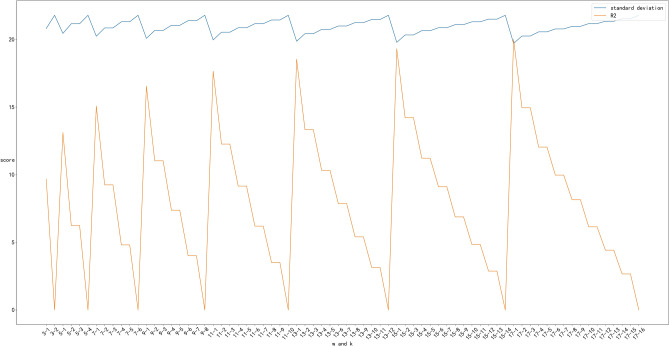
Optimal parameters of the SG algorithm.

To obtain a good SG filtering effect, a sensitivity analysis of the SG algorithm is carried out, the smoothing effect of the SG algorithm is performed on the data, and the filtering effects of the window length and polynomial order are compared; these two aspects are considered. One is to try to maintain a smoothing effect, that is, to use the standard deviation of the field data and the smoothed data to ensure that the dispersion degree of the data is better, and the other is to compare R^2^ to ensure that the smoothed data are as good as possible and that the field data are consistent. The window length was set from 3 to 29 for experimental comparison:

As shown in Fig. 3, the best filtering parameters are 17 and 1 because R^2^ is close to 1 and the data standard deviation is relatively small, resulting in better data noise reduction. Preprocessing via the SG algorithm, clearly revealed that the data quality is better, and some abnormal points are removed more smoothly, which is more in line with the actual drilling situation.

**Figure 4:**
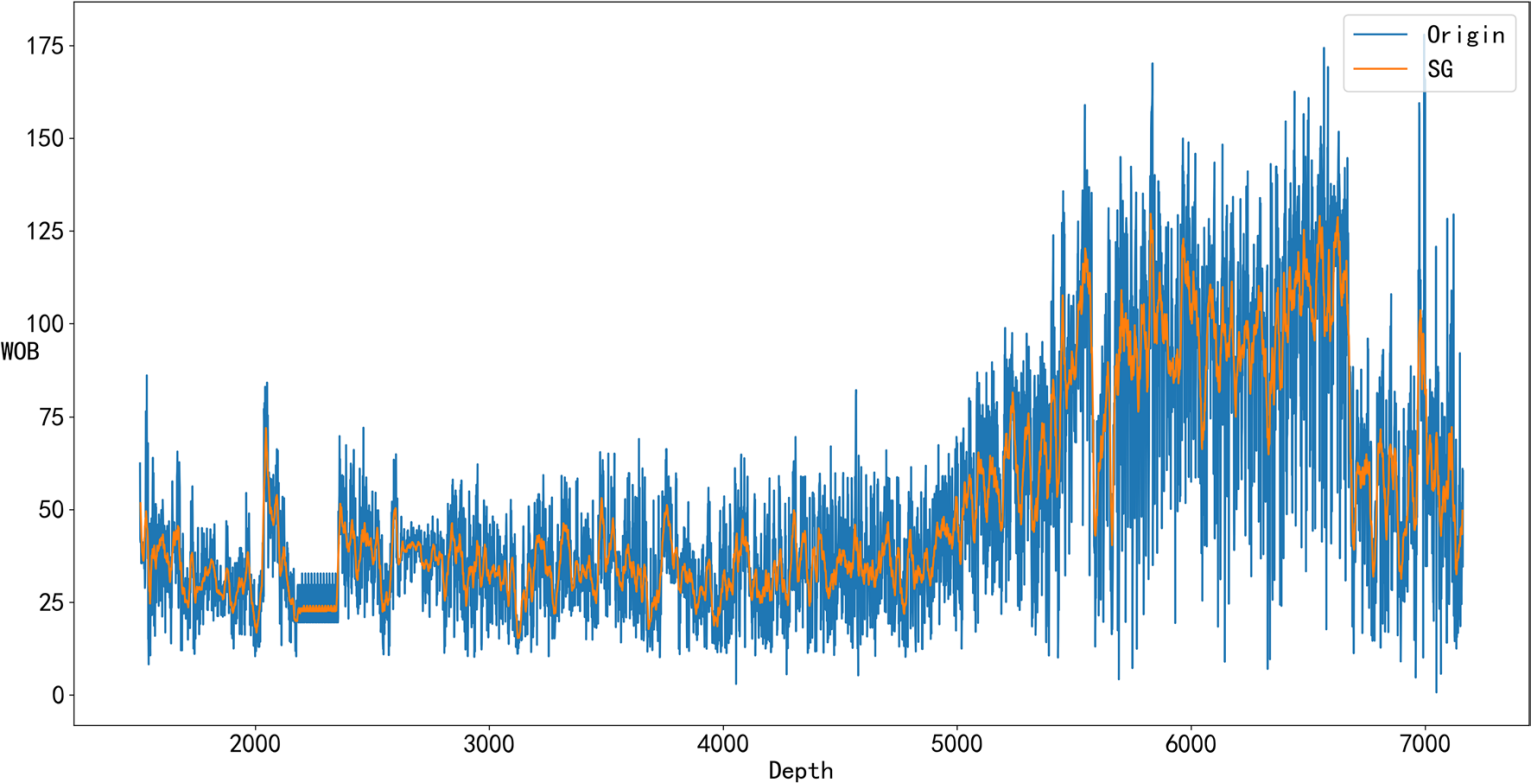
Comparison of the SG denoised and original WOB.

**Figure 5:**
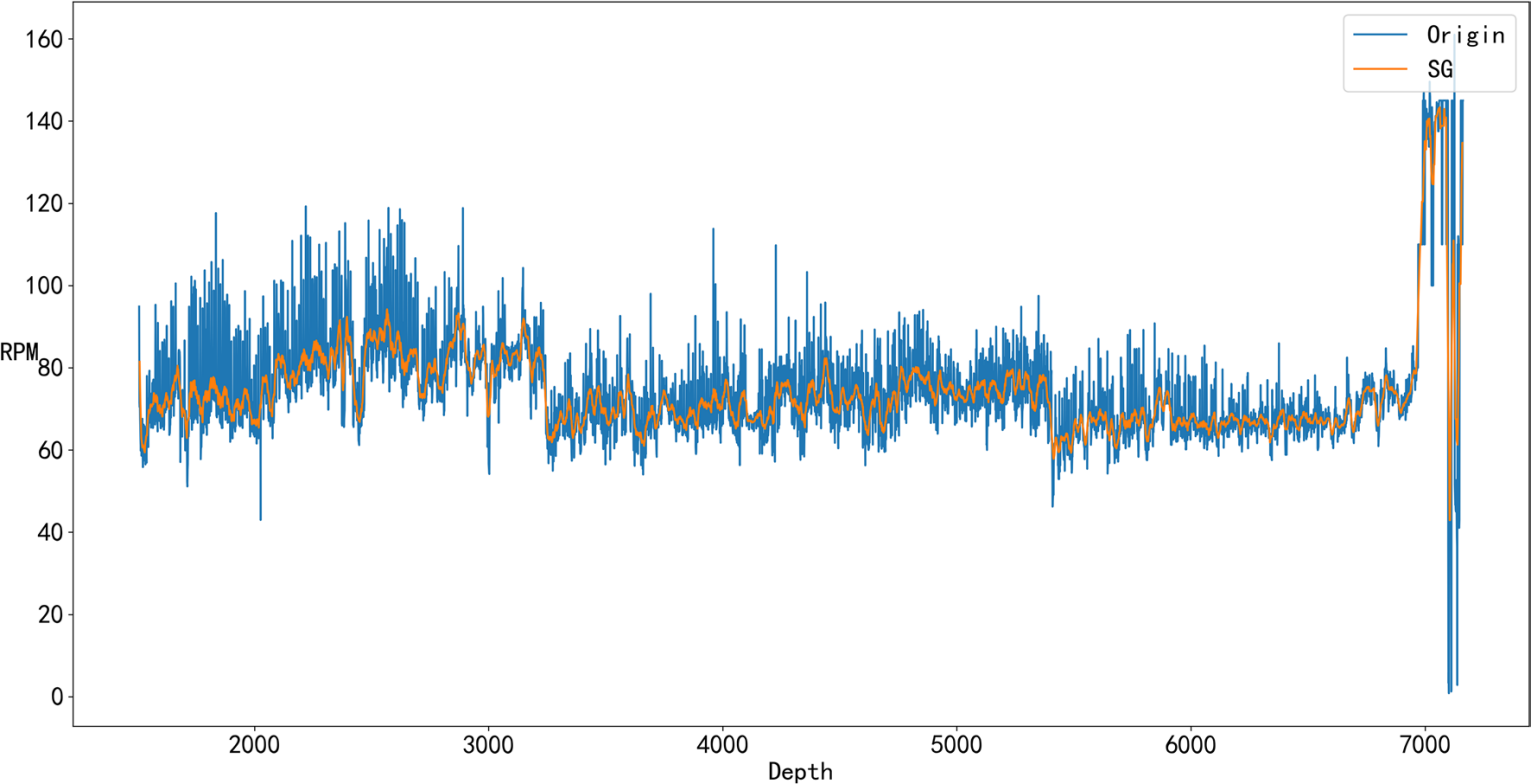
Comparison of the SG denoised and original RPM.

**Figure 6:**
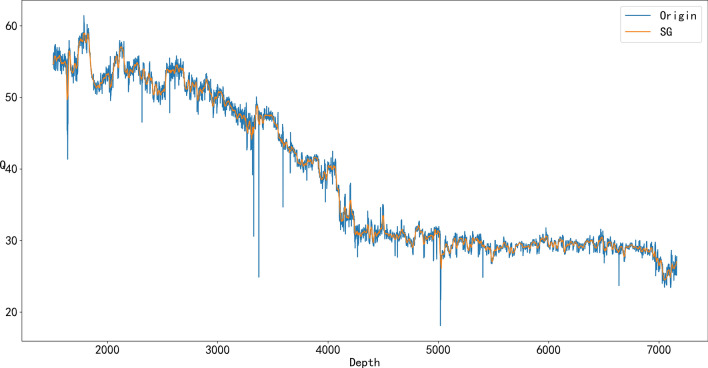
Comparison of the SG denoised and original Q.

**Figure 7:**
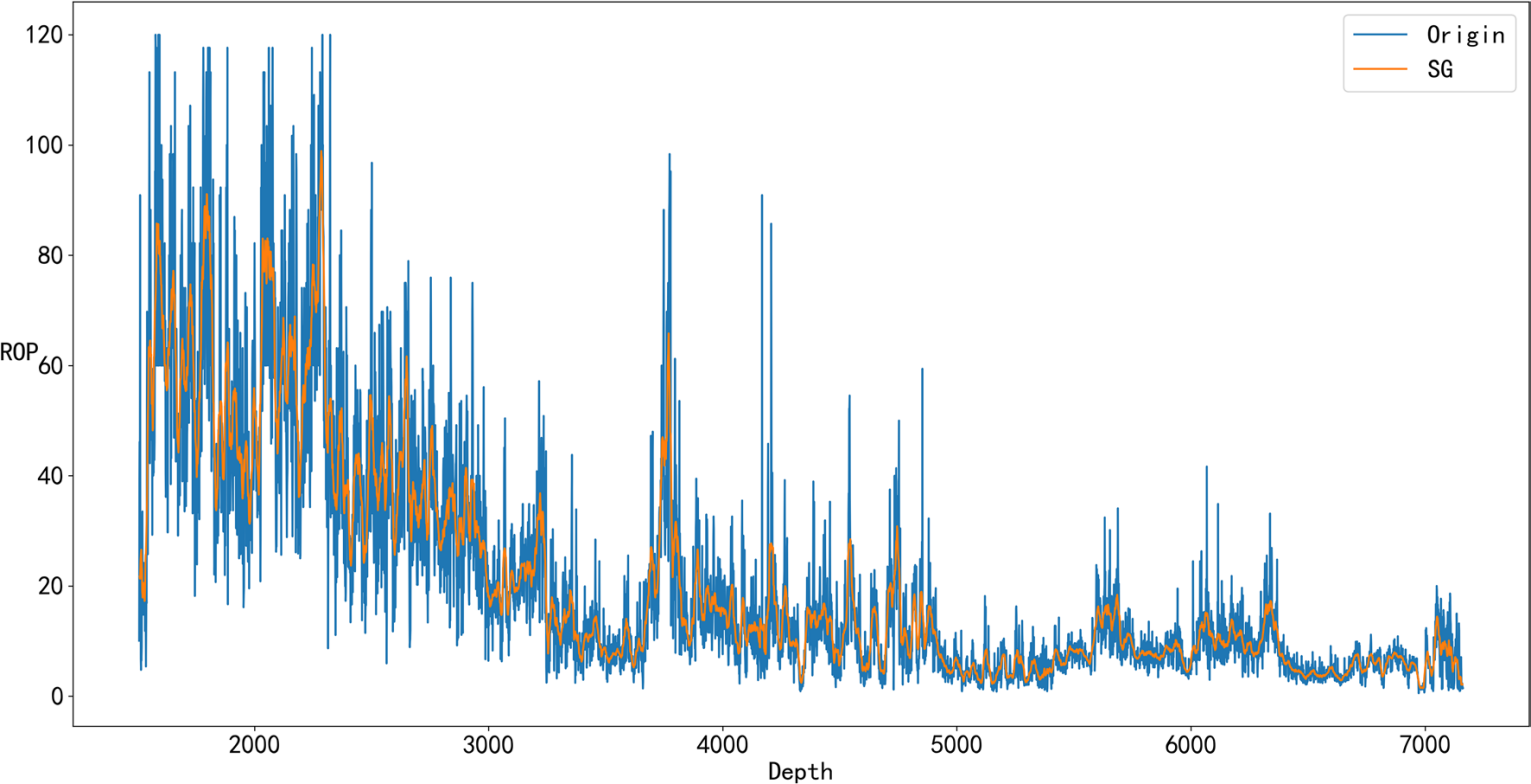
Comparison of the SG denoised and original ROP.

From Figs. 4, 5, 6, and 7, it can be seen that denoising is necessary to ensure that the impact of various abnormal situations can be removed and the data more in line with the real situation can be obtained. For example, in Fig. 6, Q has a dozen exceptions that were filtered by the SG algorithm so that the overall trend of the Q data is more consistent with the actual drilling conditions.

### Data spilt

According to the drilling field, different drilling bits are suitable for different well sections and formations. To avoid the influence of different kinds of bits, the drilling data from the drilled well were divided into nine datasets, namely, the whole well dataset, first bit dataset, second bit dataset, third bit dataset, fourth bit dataset, fifth bit dataset, sixth bit dataset, seventh bit dataset and eighth bit dataset according to the type of bit used; the specific segments are shown in Table 3.

Whether through the regression fitting of physical models or during the training process of ML models, how to divide the training dataset and test dataset has a great impact on the prediction effect. Moreover, during the drilling process in the drilling field, the logging data are acquired segment by segment, and the random spilt method perhaps can’t be meet. Therefore, in this study, nine datasets all were spilt with two kinds of method including randomly divided and divided according to the ration before and after.

### Feature selection

Table 2 shows that there are approximately 18 kinds of drilling variables, which may be related to the ROP. However, some drilling variables need not be taken into account because they may not be measured directly but may be calculated twice by some of the measured variables, such as DC and DCN. In addition, some variables, such as MD, TVD and Bit Depth, have the same meaning and effect and simply consider MD.

According to the mechanistic analysis, 14 variables were retained, namely, MD, HL, RPM, DT, ROP, WOB, TORQUE, SPP, Q, ECD, ${d}_{b}$, $\mu$, ${G}_{f}$ and ${g}_{p}$. However, to quantitatively analyze the relationship between each variable and the ROP, correlation analysis was carried out. Common methods for correlation analysis include Pearson, Spearman and Kendall correlation analyses, which quantify correlations by correlation coefficient. However, in statistics, the Pearson correlation coefficient (PCC) is a correlation coefficient that measures linear correlation between two sets of data^41^; Spearman’s rank correlation coefficient is a nonparametric measure of rank correlation (statistical dependence between the rankings of two variables)^42^; and the Kendall rank correlation coefficient is a statistic used to measure the ordinal association between two measured quantities^43^. The three correlation analysis methods generally need to follow a linear or normal distribution or order, and are not suitable for variable correlation related to ROP.

**Figure 8:**
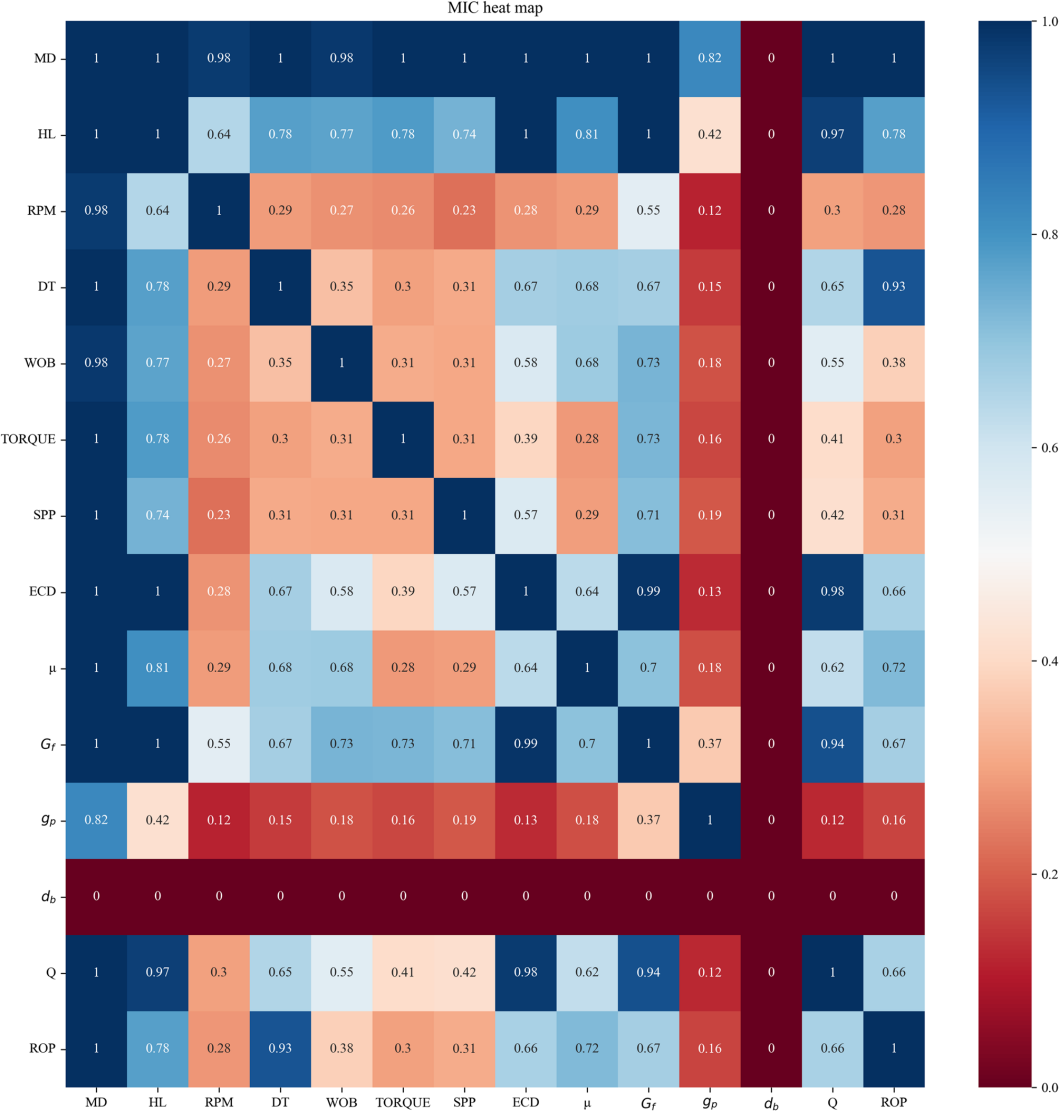
Maximal information coefficient results of the drilling data reveal the correlations between the variables: the larger the coefficient is, the greater the correlation is; the smaller the coefficient is, the smaller the correlation is.

The maximal information coefficient (MIC)^44^, which was proposed in 2011, has two excellent properties: generality and equitability. In other words, the MIC can detect various relationships including linear, non-linear, functional and non-functional relationships. The MIC values of the different types of relationships are similar at the same noise level^45^. Therefore, in this study, the MIC was selected as the correlation analysis method. The correlation heat map obtained from the MIC is shown in Fig. 8. Variables with high correlation can be used as references for feature selection and modeling.

### Physical models

In 1965, Bingham proposed an early R–W–N form of an ROP prediction model considering the influence of drill bit diameter. An empirical coefficient b is added to the ratio of the drilling pressure to the diameter of the drill bit, and a comprehensive drilling coefficient a is also included, which is calculated and fitted using the field data of each formation^3^. The specific model is as follows:

1 $${\text{ROP}}={\text{a}}{\left(\frac{WOB}{{d}_{b}}\right)}^{b}RPM$$

where a and b are dimensionless coefficients of experience corresponding to each formation, WOB is the weight on the drilling bits (KN), d_b_ is the diameter of the drill bit (mm), and RPM is the bit revolution per minute (rev/min). Although both model coefficients (a and b) were determined for the entire rock formation, they could not account for changes in the physical behavior of the well within different operating parameter areas^7^.

In 1967, Eckel studied the influence of the drilling fluid flow rate and hydraulic parameters on drilling speed through the indoor laboratory of micro drill bits, and proposed a drilling speed prediction equation that includes the drilling fluid flow rate on the basis of the drilling speed equation proposed by traditional experiments^4^.

2 $${\text{ROP}}={\text{K}}*{WOB}^{a}{RPM}^{b}{\left(\frac{kQ\rho }{d\mu }\right)}^{c}$$

3 $${\text{ROP}}={\text{K}}*{WOB}^{a}{RPM}^{b}$$

where K is the comprehensive dimensionless coefficient of drilling; a, b and c are dimensionless coefficients; WOB is the drilling pressure; RPM is the turntable speed; Q is the drilling fluid flow rate (L/s); $\uprho$ is the specific gravity of the drilling fluid (dimensionless density); d is the nozzle diameter; $\upmu$ is the drilling fluid viscosity; and k is the dimensionless coefficient fitted by the microdrill laboratory and field data.

Although Eq. (2) describes the relationship between the properties of the drilling fluid and the ROP to a certain extent, in actual drilling operations, the density and viscosity of the drilling fluid are designed in advance and generally do not change during the actual drilling process. The operating parameters that are convenient for driller adjustment and control on site are the WOB, RPM and Q. Considering the actual working conditions, Eckel’s drilling speed equation is simplified, and a simplified Eckel drilling speed prediction equation is proposed, as shown in Eq. (3).

In 1974, Bourgoyne and Young^6^ proposed a broad ROP model that included all common drilling parameters. Subsequently, in 1986, the newly proposed modified Bourgoyne and Young formula eliminated normalization factors and constant parameters^46^, simplifying the model and making it dependent on the measurement of core variables in real-time drilling optimization:

4 $$\frac{dD}{dt}=Exp\left({a}_{1}+{\sum }_{j=2}^{8}{a}_{j}{x}_{j}\right)$$

5 $${\text{ROP}}=\left({f}_{1}\right)\left({f}_{2}\right)\left({f}_{3}\right)\left({f}_{4}\right)\left({f}_{5}\right)\left({f}_{6}\right)\left({f}_{7}\right)\left({f}_{8}\right)$$

$${f}_{1}={e}^{2.303{a}_{1}}=K$$

$${f}_{2}={e}^{2.303{a}_{2}(10000-D)}$$

$${f}_{3}={e}^{2.303{a}_{3}{D}^{0.69}\left({g}_{p}-9.0\right)}$$

$${f}_{4}={e}^{2.303{a}_{4}D({g}_{p}-{\rho }_{c})}$$

$${f}_{5}={\left(\frac{\frac{WOB}{{d}_{b}}-{(\frac{WOB}{{d}_{b}})}_{t}}{4.0-{(\frac{WOB}{{d}_{b}})}_{t}}\right)}^{{a}_{5}}$$

$${f}_{6}={(\frac{RPM}{100})}^{{a}_{6}}$$

$${f}_{7}={e}^{-{a}_{7}h}$$

6 $${f}_{8}={(\frac{{F}_{j}}{1000})}^{{a}_{8}}$$

Although Bourgoyne and Young’s mechanical ROP model provides a comprehensive description of the drilling process, many of the parameters used in the model are difficult or impossible to measure in real time with prior art and must be approximated, such as the pore pressure gradient and drill bit wear. In addition, the model relies on normalization constants for drill bit depth and the WOB, RPM and Q terms, first derived from the 1970s. Nascimento^47^ and Kutas^48^ exposed this problem by reporting Bourgoyne and Young model applications by different authors with different normalization factors and proposing new values.

In 2019, Cesar Soares proposed a new drilling speed model^7^ based on real-time drilling speed prediction and the problems of Bourgoyne and Young. The validity of the coefficients can be guaranteed only if the model predicts the exact same bit, drilling fluid, formation and mechanical drilling speed under similar operating conditions. Therefore, in a certain range of drill bits or mud, the same coefficient values are not applicable, and one model coefficient can absorb the influence of all constant parameters. The newly proposed modified Bourgoyne and Young formula eliminates the normalization factor and constant parameters, simplifying the model so that it relies on the measurement of core variables in real-time drilling optimization.

7 $${\text{ROP}}={a}_{1}{D}^{{a}_{2}}{WOB}^{{a}^{5}}{RPM}^{{a}^{6}}{q}^{{a}_{8}}$$

### ML models

#### Artificial neural network

**Figure 9:**
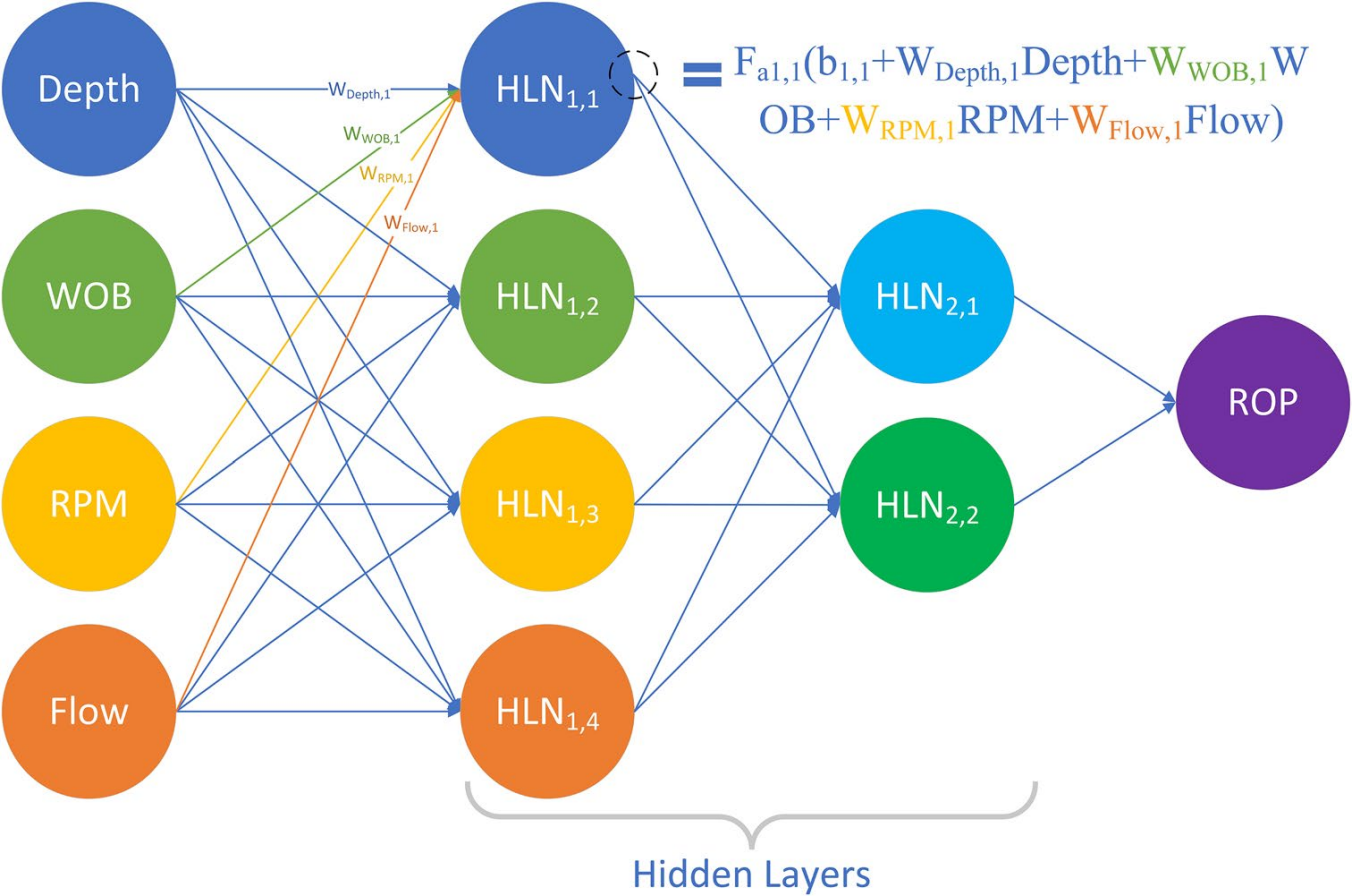
ROP model prediction structure based on ANN.

In 1997, Bilgesu introduced neural networks to train a new ROP prediction model, which was well applied in real situations^11^. The structure of ANN is shown in Fig. 9.

#### Support vector machine

**Figure 10:**
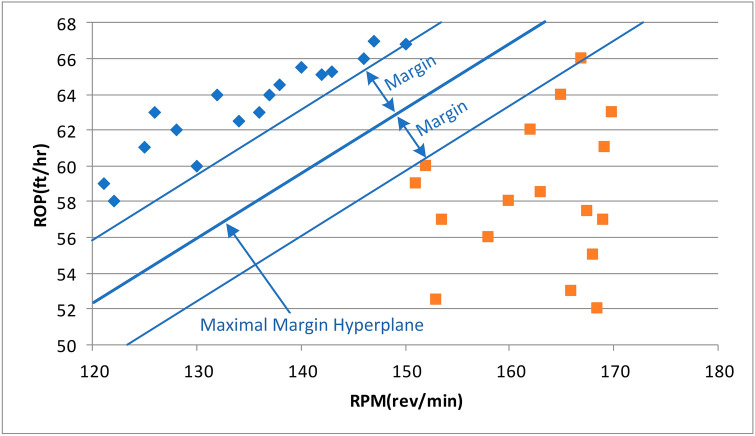
The example of SVM.

In 2015, Bodaghi proposed an “optimized support vector machine regression” method for ROP prediction^18^. Optimizing the parameters of the support vector machine by optimizing the parameters of the cuckoo search algorithm has a higher ROP prediction accuracy than the genetic algorithm and the pattern search and grid search algorithms. The largest edge hyperplane is based entirely on the data point located at the edge. These two points, equidistant from the hyperplane, are called support vectors. Figure 10 shows an ideal example of detecting drilling speed anomalies. Given the additional drilling parameters and actual site conditions, it is unreasonable to expect linearly separable data to distinguish between efficient drilling and high vibration, inadequate hole clearing, or drill bit ladle operation areas. Support vector machines overcome this problem by allowing some data points to violate margins.

#### Random forest

**Figure 11:**
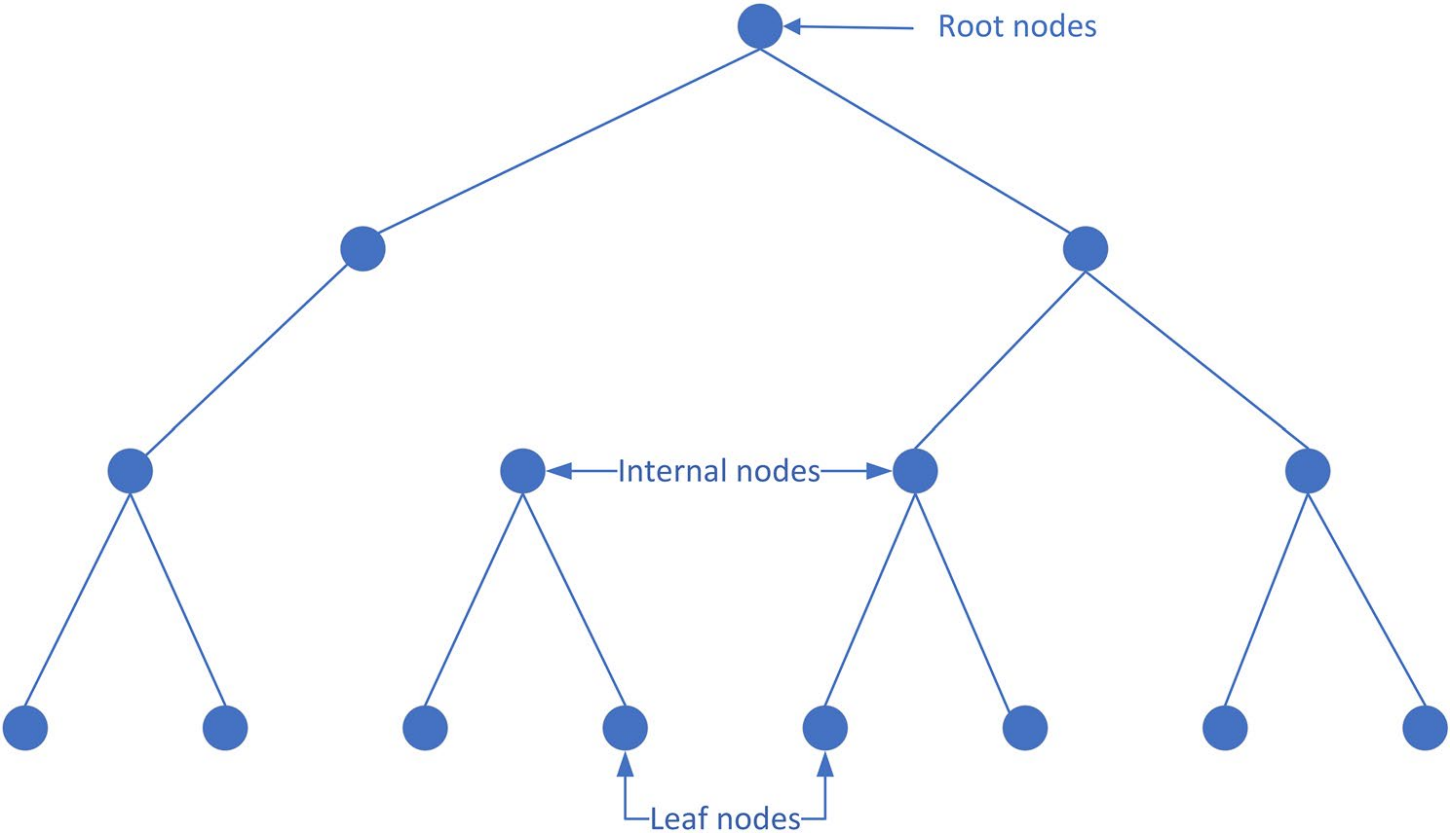
The structure of RF.

In 2015, Hegde used limited ground measurements to predict ROP using Trees, Bagging, and Random Forest, respectively, and random forests provided the best accuracy for the data used; therefore, real-time, closed-loop applications were used^19^. Different regression algorithms, including Least Squares Regression, Ridge and Lasso Regression, and Principal Components Regression, have also been used to predict ROP incidence. The random forest algorithm is a Bagging ensemble algorithm based on decision trees^49^. The structure of the random forest algorithm is shown in Fig. 11. The calculation relies mainly on the construction of multiple decision trees, and the average value of each decision tree is taken as the final prediction result. The original sample sampling of each decision tree is random, and the sampling processes are independent of each other. The specific training process of the random forest regression algorithm is shown in Fig. 11.

1. Randomly put back the sampling to construct the original samples X and Y of the decision tree, and construct the root node of the decision tree.
2. Calculate the feature number m of the original sample X and the size n of the training set.
3. If m < 1, the training is over. If m ≥ 1 iterates through all the values of m features on the training set, each value is used as the segmentation point, the impurity of different features of each segmentation point is calculated, and the feature with the smallest impurity is the segmentation feature of the segmentation point. then M-1; Repeat this step until the end of training. The prediction output is the average of all sample values for the current node sample set.
4. Repeat (1) (2) (3) until all decision trees have been trained. Output averages for all decision trees.

### Model performance evaluation metrics

Whether it is a classical physical model or an ML model, it is essentially a fitting of the real drilling law, and the effect of its fitting needs to be evaluated and measured by certain indicators. Common model performance evaluation indicators are selected: the root mean square error (RMSE), average absolute percentage error (MAPE) and coefficient of determination (R^2^). The RMSE is the square root of the square of the deviation between the predicted value and the true value and the ratio of n observations; this metric measures the deviation between the predicted value and the true value, and is sensitive to outliers in the data.

8 $${\text{RMSE}}=\sqrt{\frac{1}{{\text{N}}}\sum_{{\text{i}}=1}^{{\text{n}}}{\left({{\text{ROP}}}_{{\text{Field}},\mathrm{ i}}-{{\text{ROP}}}_{{\text{Model}},\mathrm{ i}}\right)}^{2}}$$

where N is the number of samples, ${{\text{ROP}}}_{{\text{Field}},\mathrm{ i}}$ represents the actual drilling speed at the site and ${{\text{ROP}}}_{{\text{Model}},\mathrm{ i}}$ represents the drilling speed predicted by the drilling speed model.

The MAPE, which measures the relative error of a predicted value to a true value, is one of the most popular metrics for evaluating forecast performance, as defined below:

9 $${\text{MAPE}}=\frac{1}{{\text{N}}}\sum_{{\text{i}}=1}^{{\text{n}}}\frac{|{{\text{ROP}}}_{{\text{Field}},\mathrm{ i}}-{{\text{ROP}}}_{{\text{Model}},\mathrm{ i}}|}{{{\text{ROP}}}_{{\text{Field}},\mathrm{ i}}}\times 100\mathrm{\%}$$

The R^2^ evaluates the fitting performance of the regression model. When R^2^ is close to 1, the model has good prediction accuracy.

10 $${R}^{2}=1-\frac{\sum_{{\text{i}}=1}^{{\text{n}}}{\left({{\text{ROP}}}_{{\text{Field}},\mathrm{ i}}-{{\text{ROP}}}_{{\text{Model}},\mathrm{ i}}\right)}^{2}}{\sum_{{\text{i}}=1}^{{\text{n}}}{\left({{\text{ROP}}}_{{\text{Model}},\mathrm{ i}}-\frac{1}{{\text{N}}}\sum_{{\text{i}}=1}^{{\text{n}}}{{\text{ROP}}}_{{\text{Field}},\mathrm{ i}}\right)}^{2}}$$

### Hyperparameter selection in ML models

The hyperparameters control the structure of ML models and determine their performances, which are parameters that are not directly learnt within training process. There are no definite rules for hyperparameter selection, as optimal model structure varies by application. Each ML model has multiple different hyperparameters. For instance, the hyperparameters of the RF include the number of trees in the forest (n_estimators), the function to measure the quality of a split, the number of features to consider when looking for the best split (max_features) and the minimum number of samples required to be at a leaf node (min_samples_leaf). The number of trees in the forest and the minimum number of samples required to be at a leaf node are closely related to the training accuracy of the RF model. The more n_estimators and min_samples_leaf, the more accurate the prediction performance is; however, the training costs of the RF increase. Therefore, it is necessary to balance the relationship between training accuracy and training costs. Researchers typically define a grid and search for the best hyperparameter combinations with cross-validation. The same methodology was applied in this study, and it was implemented using the sklearn.model_selection.GridSearchCV function of Python’s scikit-learn^50^.

**Figure 12:**
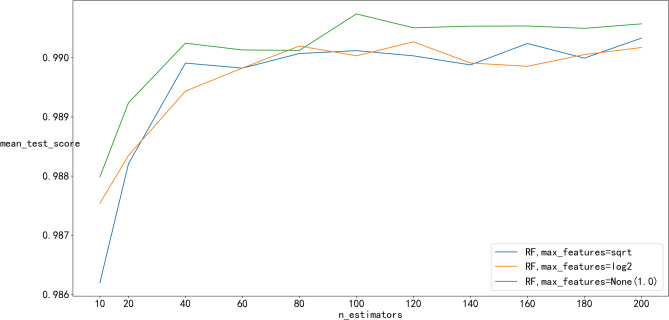
The process of grid search CV.

After referring to the hyperparameters in Sepideh^51^ and several cross-validations, the optimal hyperparameters of those ML models selected are shown in Table 4. To provide a more specific explanation of the process of hyperparameter selection, the optimization process of two RF hyperparameters, n_estimators and max_features, is illustrated in Fig. 12. As shown in Fig. 12, the mean_test_score of max_features = None(1.0) is the highest among the three max_features, and the mean_test_score is the average test core obtained from fivefold cross validation so that overfitting can be avoid in the process of hyperparameter selection. Among the n_estimators values, n_estimators = 14 had the highest mean_test_score, and max_features = None (1.0) and n_estimators = 140 were ultimately selected as the optimized hyperparameters. In this way, two hyperparameters are selected, and the hyperparameters of the other ML models are also determined in accordance with this process.

**Table 4:** Hyperparameters of ML models.

Models	Hyperparameter	Value
ANN	The ith element represents the number of neurons in the ith hidden layer	(1000, 500)
	Activation function for the hidden layer	relu
	The solver for weight optimization	adam
	Strength of the L2 regularization term	0.01
	Maximum number of iterations	500,000
SVM	Specifies the kernel type to be used in the algorithm	rbf
	Degree of the polynomial kernel function	3
	Kernel coefficient for ‘rbf’, ‘poly’ and ‘sigmoid’	scale
	Regularization parameter	50.0
	Epsilon in the epsilon-SVR model	0.1
RF	The number of trees in the forest	140
	The function to measure the quality of a split	SE
	The number of features to consider when looking for best split	1.0
	The minimum number of samples required to be at a leaf node	2

### Hybrid physics-ML models

The purpose of ROP prediction is to better guide the optimization of drilling parameters, and three physical data models including WOB, RPM and Q are selected from the actual application situation on site, namely, the physical model proposed by Bingham in 1965 (Eq. 1), the modified physical model proposed by Eckel in 1967 (Eq. 3) and the physical model proposed by Soares in 2019 (Eq. 7). With a certain amount of logging data, the optimize method in Python’s SciPy library is used for multivariate nonlinear fitting to obtain the empirical coefficients of the three physical models.

Three common ML algorithms were selected—ANN, SVM, and RF as the learning model. This study proposes four approaches of hybrid physics-ML modeling.

### Residual modeling

**Figure 13:**
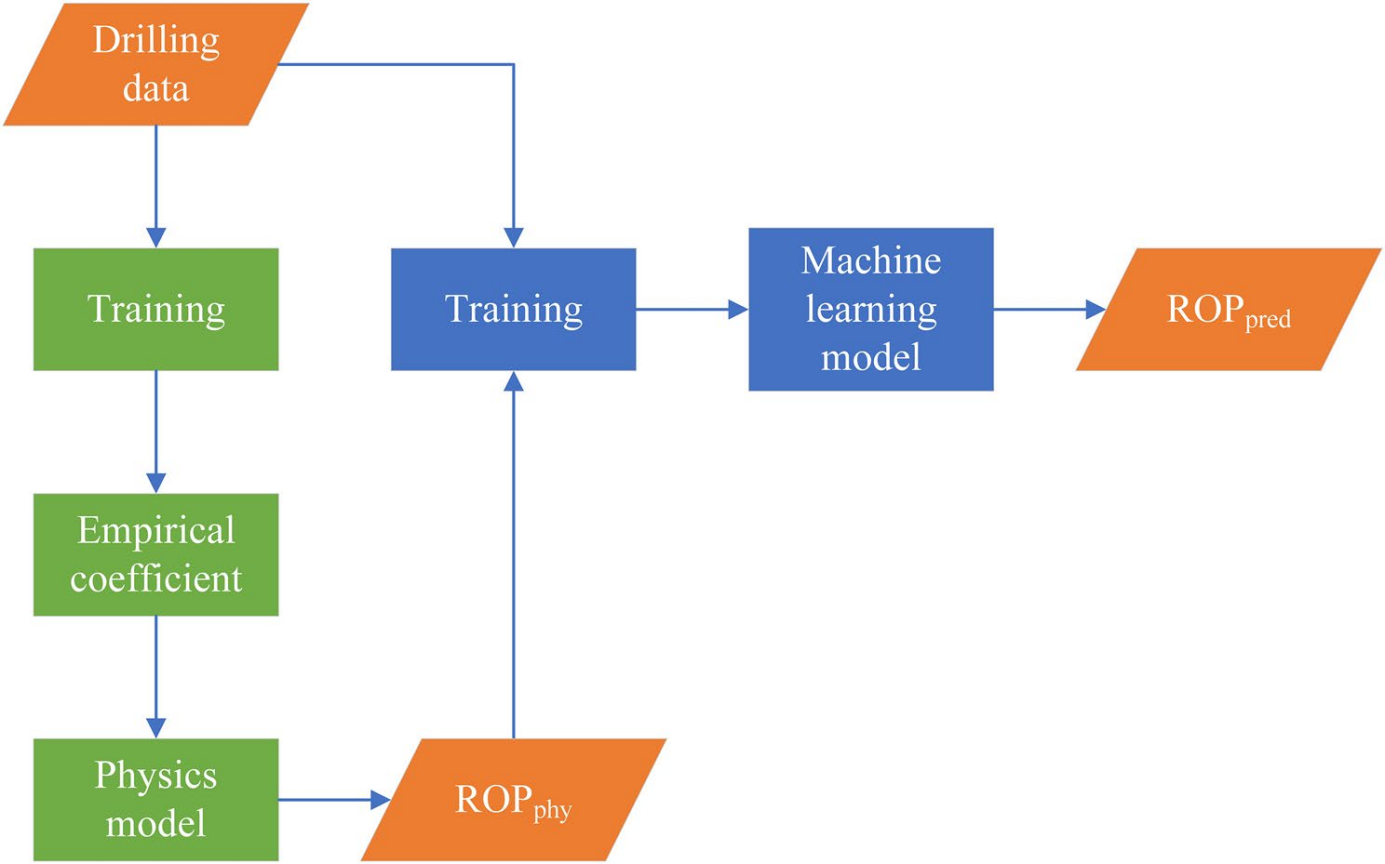
The structure of residual modeling.

The residual approach may be the oldest and most common approach for directly addressing the imperfection of physics-based models in the scientific community; an ML model (usually linear regression) learns to predict the errors or residuals made by a physics-based model^52^. The structure of the residual model is shown in Fig. 13. First, drilling data are used to train the physics model with a regression method to obtain the empirical coefficients of the physics model. Then, the physics model can obtain the ${ROP}_{phy}$ when the drilling data are the input of the physics model. Finaly, the drilling data and ${ROP}_{phy}$ are used as the inputs of the ML model which is subsequently trained on a hybrid residual model.

### Integrated coupling

**Figure 14:**
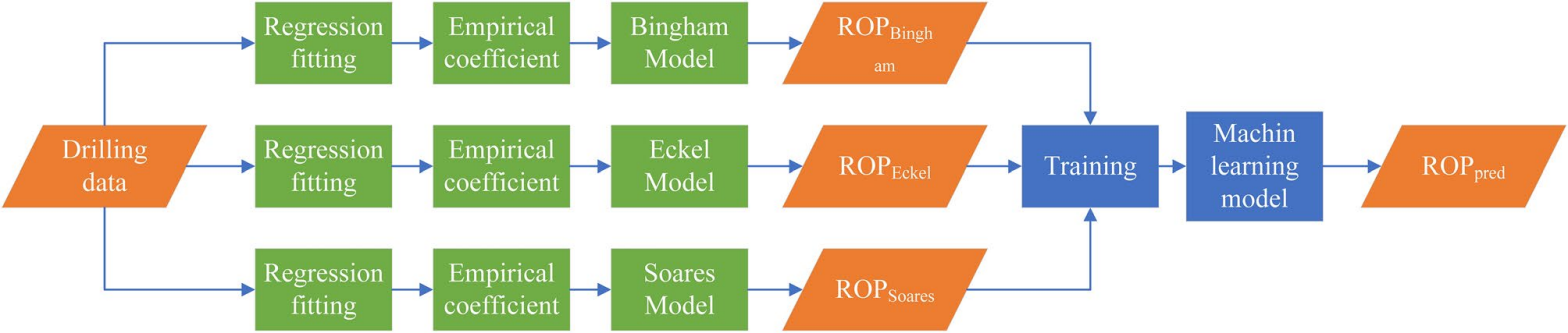
The structure of integrated coupling.

Different physical ROP models have different emphases, and the ML model can combine their different advantages when the output of the physical model is used as input to the ML model. A detailed description of this approach of hybrid modeling is shown in Fig. 14. First, the drilling data are used for three physical model regression fitting. Then, the output from Bingham model, Eckel model and Soares model are recognized as inputs to the ML model to train the ML model, which may yield better prediction than individual physics models.

### Simple average

**Figure 15:**
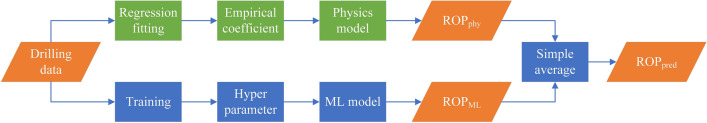
The structure of simple average.

Physical models have better generalization performance and interpretability than ML models, but most of ML models have better accuracy when enough good data are available for training ML models. When predictions from both physics and the ML model are combined, the prediction of the ROP may improve. In this study, the simple average is the combined approach, which is shown in Fig. 15. First, the physics model and ML model are regression fit and trained alone to obtain predictions from both the physics model and the ML model. Then, the average of ${ROP}_{phy}$ and ${ROP}_{ML}$ is recognized as the final prediction of the ROP from the hybrid model.

### Bagging

Ensemble learning works by building and combining multiple learners to accomplish learning tasks; this approach is sometimes referred to as multi-classifier system or as committee-based learning^53^. By combining multiple learners, ensemble learning often achieves significantly better generalization performance than does single learner learning by creating and selecting good and different learners. According to the methods used to generate individual learners, the current ensemble learning methods can be roughly divided into two categories: serialization methods with strong dependency between individual learners, which must be generated serially; and parallel methods with no strong dependency between individual learners, which can be generated simultaneously. The former is represented by Boosting and the latter is represented by Bagging and Random Forest. Because the physics prediction ROP models are not strongly dependent, this study selects Bagging as the ensemble learning method and different physics models are recognized as individual learners.

**Figure 16:**
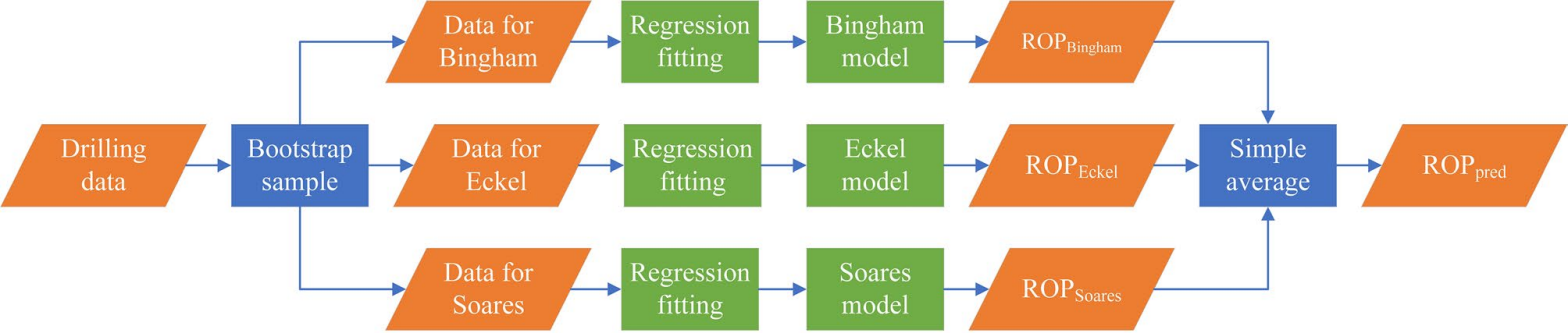
The structure of Bagging.

Bagging is a type of parallel ensemble learning^54^, that is directly based on bootstrap sampling^55^. The specific process is shown in Fig. 16. First, the drilling data are divided into three training sets for physics models through bootstrap sampling and every training set accounts for approximately 63.6% of the drilling data without repetition. Then, physics models can obtain the first prediction including ${ROP}_{Bingham}$, ${ROP}_{Eckel}$ and ${ROP}_{Soares}$, which are used for simple average to improve ${ROP}_{pred}$.

## Results and discussion

In this study, the Bingham, Eckel, and Soares models were selected as physical models for ROP prediction. Among the ML models, ANN, SVM and RF were also selected for training and testing. Three evaluation metrics, RMSE, MAPE and R^2^, were used to comprehensively compare the performances of these models, and the optimal physical and ML models were selected to establish hybrid models. Four categories of hybrid models, namely, residual modeling, integrated coupling, simple average and Bagging, were established and compared with the optimal physical and ML models. To further investigate the influence of different models on hybrid models, physical models and ML models were used to establish different hybrid models. In total, 34 kinds of drilling speed prediction models were used for a total of 2448 groups of experiments.

### Comparison of different physical models

In this section, the performances of three physical models, namely, the Bingham, Eckel and Soares models, are compared in detail, and the optimal physical model is selected. First, the coefficients in these physical models must be obtained. The Trust Region Reflective (TRF) algorithm, is motivated by the process of solving a system of equations that constitute the first-order optimality condition for a bound-constrained minimization problem, as formulated in STIR^56^. This algorithm is particularly suitable for large sparse problems with bounds and works quite robustly in unbounded and bounded problems; thus, it is chosen as the selected algorithm for solving the non-linear least squares problem to obtain the empirical coefficients contained in the physical models.

**Figure 17:**
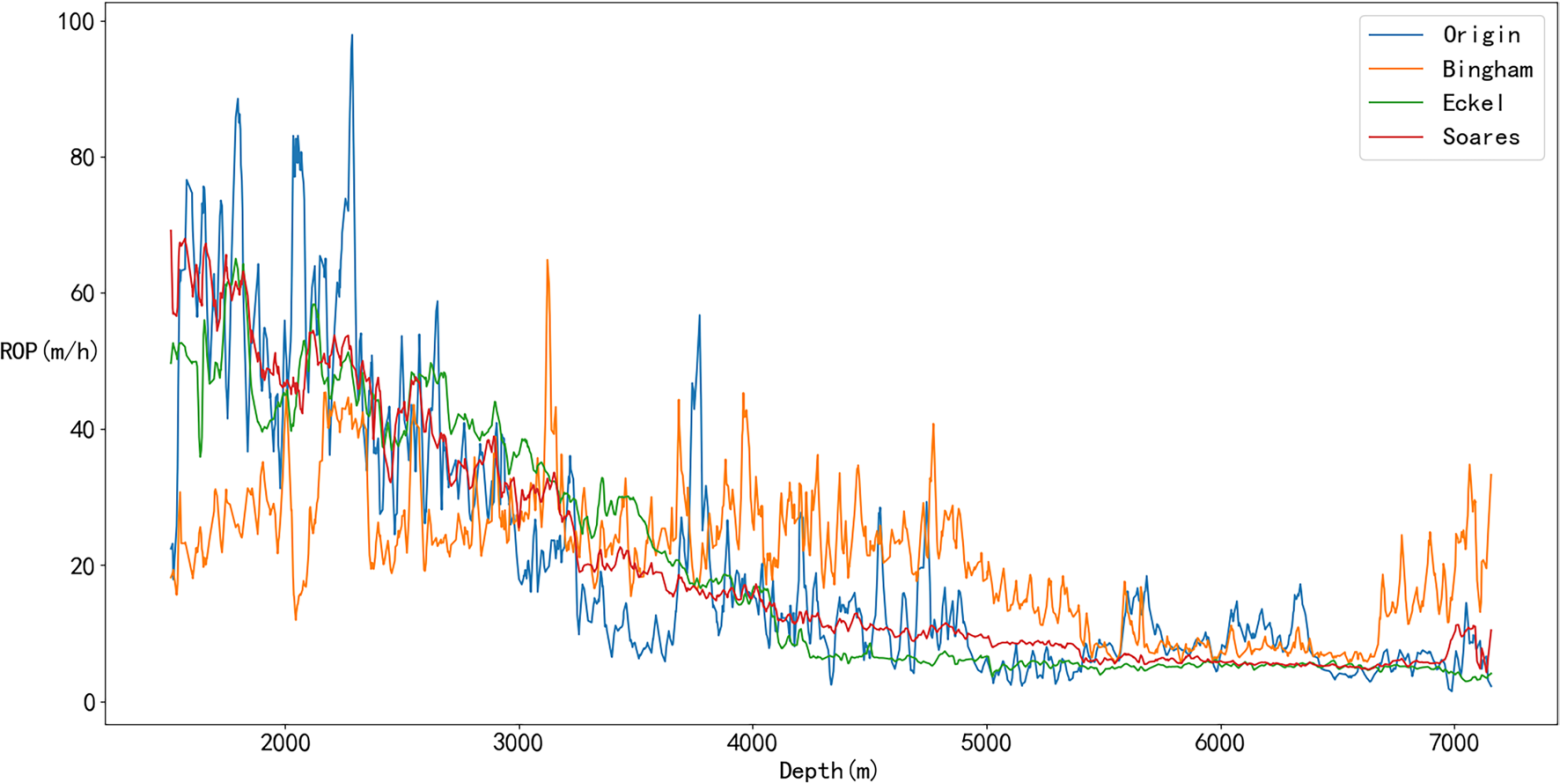
Prediction results of the physical models.

**Figure 18:**
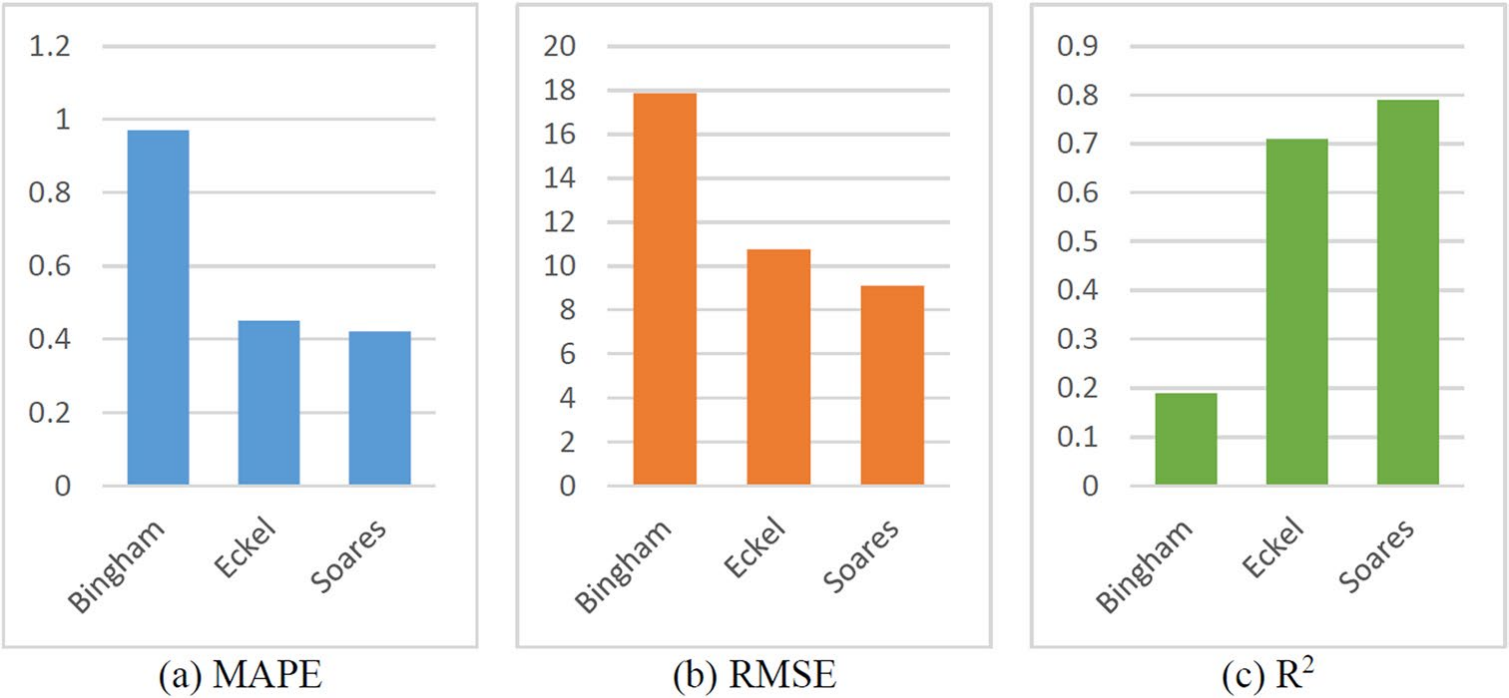
Proportionally divide the impact of different data preprocessing methods.

The performances of these three ROP prediction models are compared, and the results are shown in Figs. 17 through 18. To better reflect that the empirical coefficients obtained by the TRF algorithm can accurately predict ROP; the training set and test set are randomly divided at a ratio of 4:1, and the comparison between the ROP predicted by the three physical models and the original ROP is shown in Fig. 17. Figure 17 shows that the prediction results of the named Soares model fit the actual ROP curve more closely, confirming that this model is more suitable for this field. The R^2^ of the amended Soares model (0.7900) is larger than that of the other physical models, and its RMSE (9.0874) and MAPE (0.4190) are both smaller than those of the other physical models, indicating that the prediction accuracy of the amended Soares model is significantly better (Fig. 18 and Table 5). Therefore, this model is selected as the physical part of the hybrid part of the hybrid model for ROP prediction.

**Table 5:** Evaluation indices of the physical model results.

ROP model	MAPE	RMSE	R^2^
Bingham	0.9674	17.8656	0.1886
Eckel	0.4544	10.7523	0.7061
Soares	0.4190	9.0874	0.7900

### Comparison of different ML models

In practice, the ROP performances of different ML models are quite different for different regions, and there is no a unified model suitable for all situations. In this section, the performances of three ML algorithms, ANN, SVM and RF, were compared, and the optimal ML algorithm for the Tarim Basin was selected.

**Figure 19:**
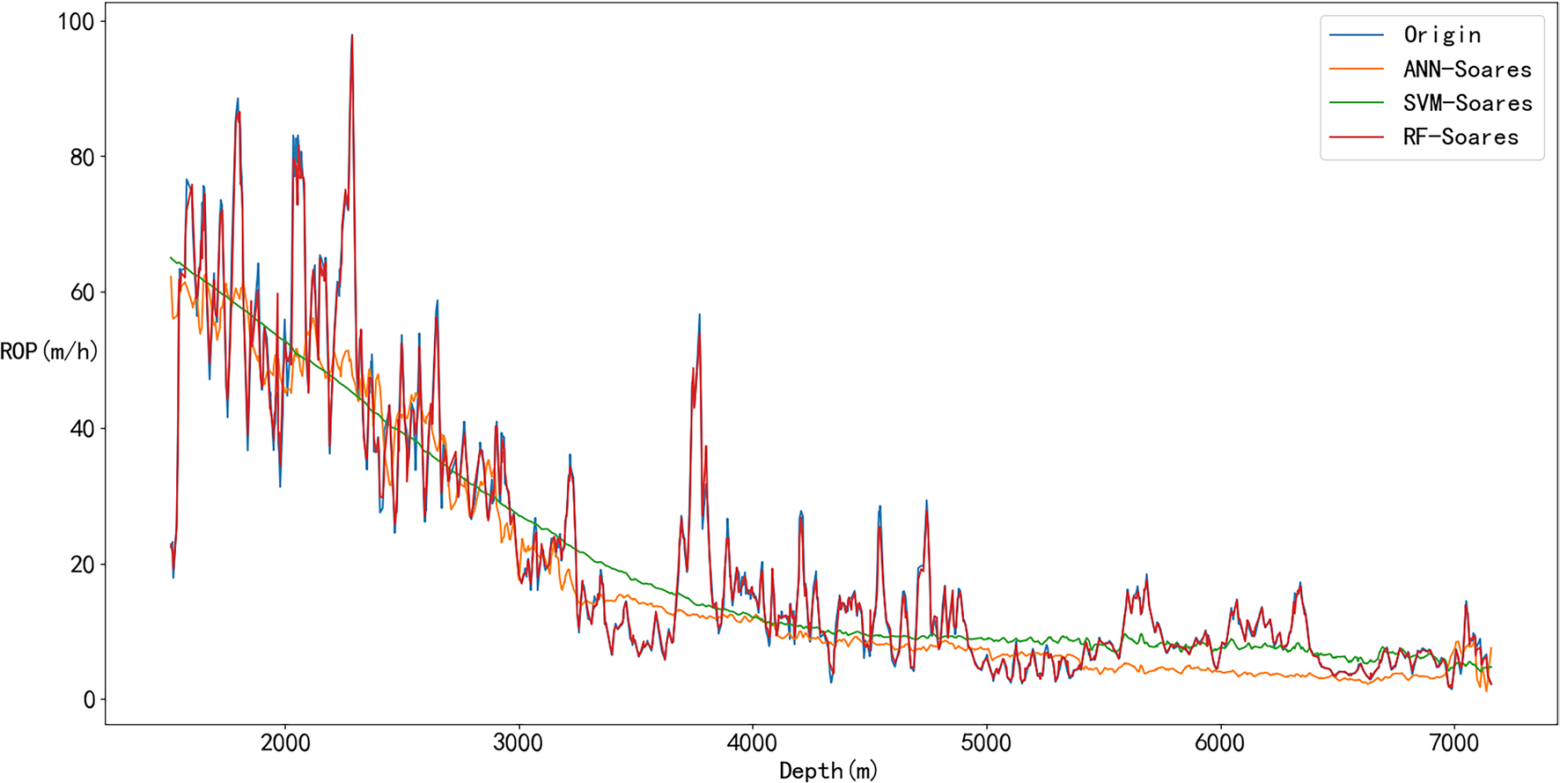
Prediction results of ML models.

**Figure 20:**
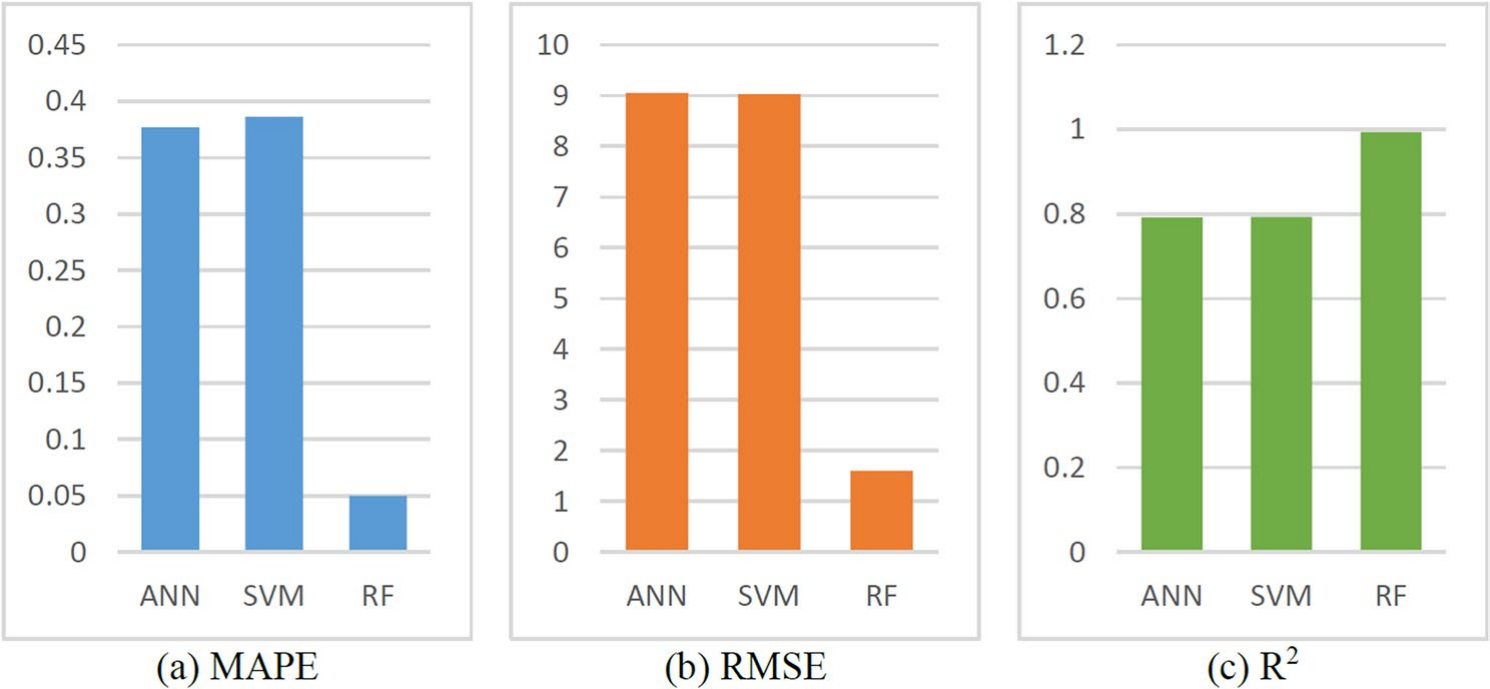
Evaluation indexes of ML model results.

The training results of the ML models are shown in Figs. 19 and 20. Due to its powerful learning capability, the prediction result of the RF model matches the original value well as shown in Fig. 19. Moreover, the training curve of the SVM also achieved a good fit. The R^2^ of the SVM prediction result is 0.7929, and the R^2^ of the RF prediction result is 0.9938 (Fig. 20 and Table 6).

**Table 6:** Evaluation indexes of ML model results.

ROP model	MAPE	RMSE	R^2^
ANN	0.3767	9.0520	0.7917
SVM	0.3862	9.0242	0.7929
RF	0.0501	1.6072	0.9934

What’s more, the RMSE and MAPE values of RF are also better than those of ANN and SVM; thus, the RF model outperforms than the other two models, and it is selected as the ML part of the hybrid model.

**Figure 21:**
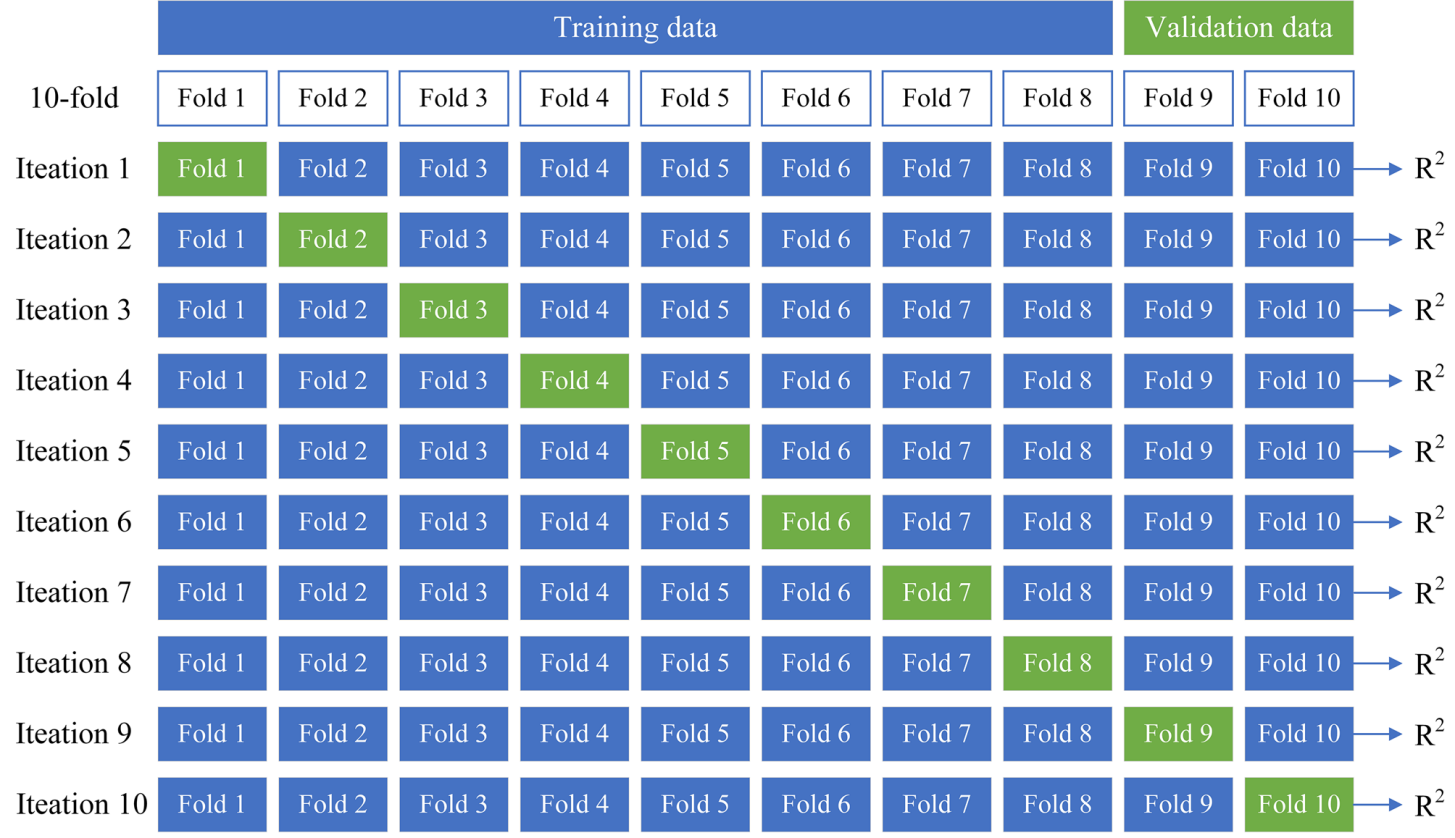
The procedure of tenfold CV.

As shown in Fig. 19 and Table 6, the RF model has a high level of ROP prediction accuracy. However, it is well known that ML models are prone to overfitting, and to avoid overfitting, this study adopts the k-fold cross-validation (CV) method to avoid overfitting and ensure generalization. With respect to the tenfold CV used in this study, the entire dataset was split into 10 smaller sets. The following procedure is followed for each of the k “folds”, as shown in Fig. 21:

A model is trained using k − 1 of the folds as training data;

The result model is validated on the remaining part of the data (it is used as a test to compute a performance measure such as accuracy).

**Figure 22:**
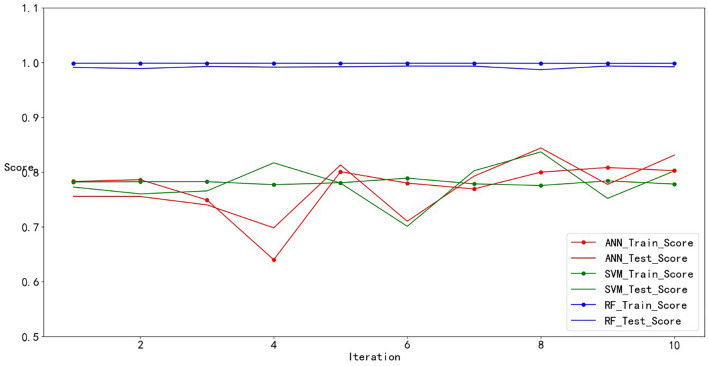
The result for tenfold CV.

The performance measure reported by k-fold CV is then the average of the values computed in the loop. It was repeated ten times until each fold was used as the validation set. A tenfold CV was performed for all three ML models, and the specific results are shown in Fig. 22.

The performance of the ML models during ten-fold CV was assessed by calculating the train score and test score, and the score represents the R^2^. According to the similar score of each fold in Fig. 22, these models can obtain similar results on different validation sets. These models are verified to have good generalization.

Furthermore, as shown in Fig. 22, there was not much difference between the train score and the test score for each cross-validation, especially for RF. It was verified that these models were not overfitted. The fluctuations in the train score and test score in the SVM and ANN models may be caused by the two models being more sensitive to fluctuations in the data. There may be different distributions between the test data and the training data, and the model may be better adapted to the distribution of the test data, resulting in higher test accuracy.

### Comparison of physical, ML, and hybrid models

The physical model with the highest prediction accuracy (Soares model) and the ML model with the best performance (RF model) were selected to construct two categories of hybrid models, including the hybrid model with residual modeling and the hybrid model with simple average. The hybrid model with integrated coupling is composed of the Bingham, Eckel, Soares and RF models. In the end, the hybrid model with Bagging included the Bingham, Eckel and Soares models.

**Figure 23:**
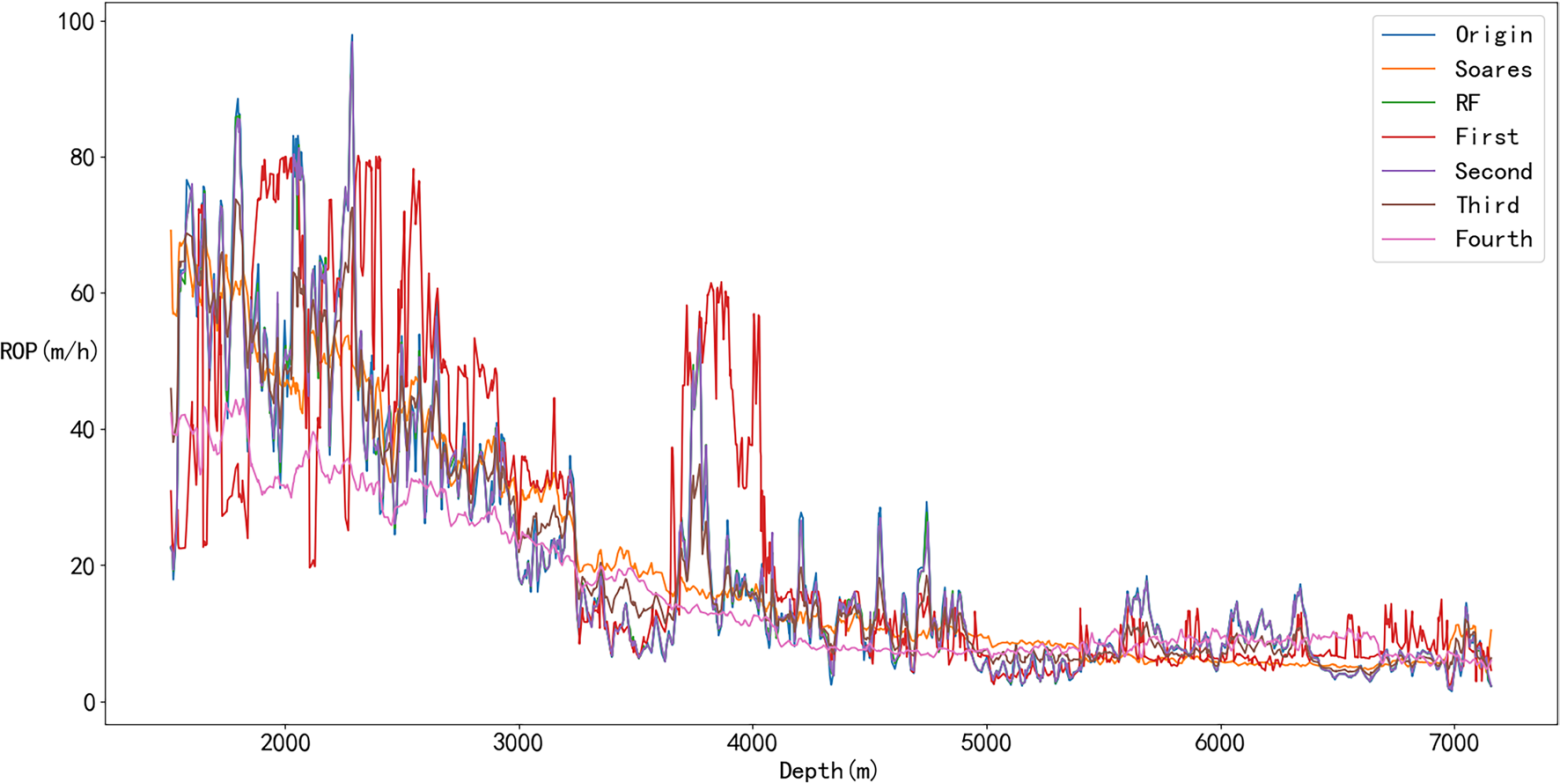
Prediction results of physical, ML and hybrid models.

**Figure 24:**
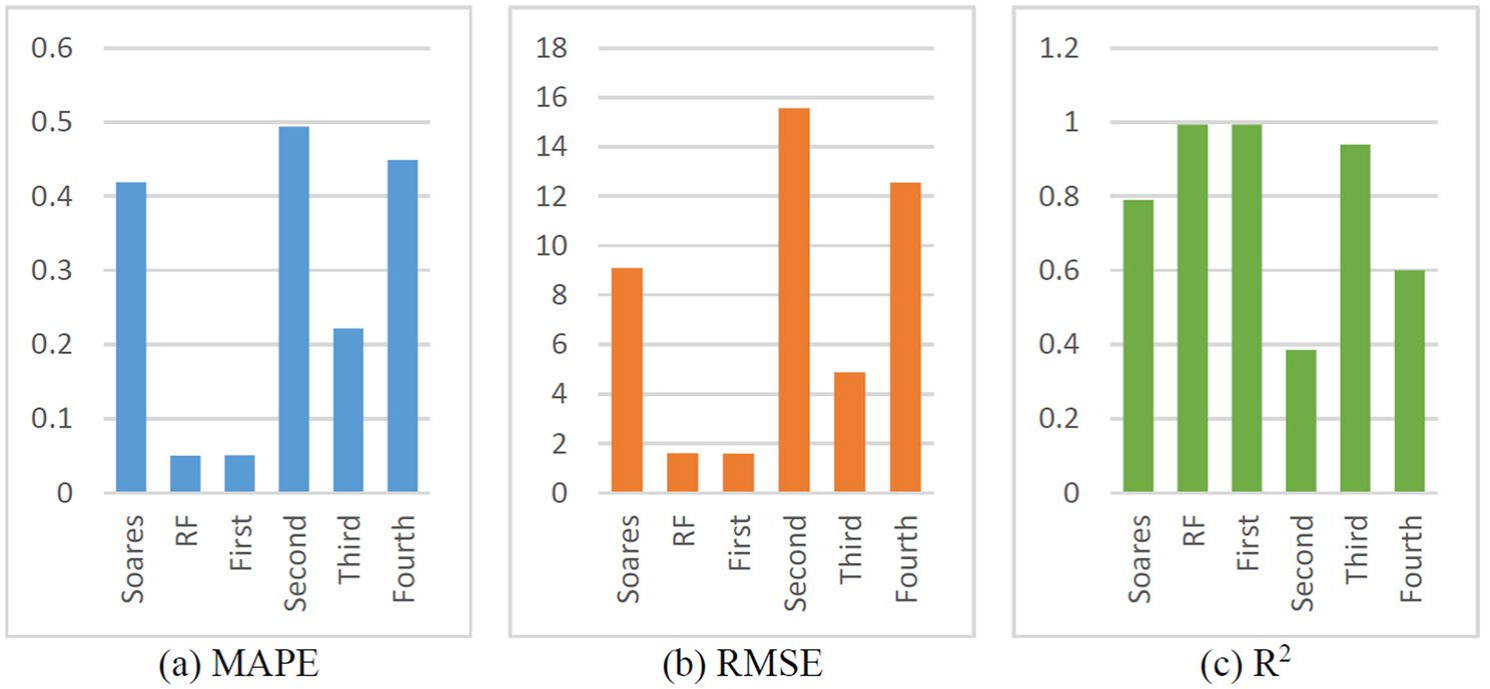
Evaluation indexes of the physical, ML and hybrid models.

The prediction results of the physical, ML, and hybrid models are shown in Figs. 23 and 24. In Fig. 23, the sign of Origin represents the ROP obtained from the drilling field, the sign of Soares represents the Soares model’s prediction, the sign of First represents the residual modeling’s prediction, the sign of Second represents the integrated coupling model’s prediction, the sign of Third represents the simple average model’s prediction, and the sign of Fourth represents the Bagging model’s prediction.

As shown in Fig. 23, Soares, Second and Fourth cannot obtain very good accurate results compared with the other models. However, when RF error compensation was introduced, the prediction results of the first hybrid model fit the actual ROP curve more closely in most well sections. According to Fig. 24 and Table 7, the prediction accuracy of the first hybrid model (R^2^ = 0.9936) is better than that of the RF model (R^2^ = 0.9934). Moreover, the prediction accuracy is also greatly improved compared with that of the Soares model. The RMSE and MAPE also exhibited better performance than did the other five models.

**Table 7:** Evaluation indices of physical, ML, and hybrid models.

ROP model	MAPE	RMSE	R^2^
Soares	0.4190	9.0874	0.7900
RF	0.0501	1.6072	0.9934
First	0.0509	1.5861	0.9936
Second	0.4939	15.5654	0.3841
Third	0.2216	4.8821	0.9394
Fourth	0.4491	12.5477	0.5998

### Comparison of different hybrid models

To further study the influence of different physical and ML models on the performance of the first hybrid model, the two best physical models (the Soares and Eckel model) and the two best ML models (SVM and RF) were selected for cross-mixing. The first model was established with the Eckel model as the ROP prediction model and the SVM as the error compensation model, and the second model was established with the Eckel model as the prediction model and the RF as the error compensation model. The third model takes the Soares model as prediction model combined with the SVM as the error compensation model, and the last model takes the Soares model as the prediction model and uses the RF as the error compensation model.

**Figure 25:**
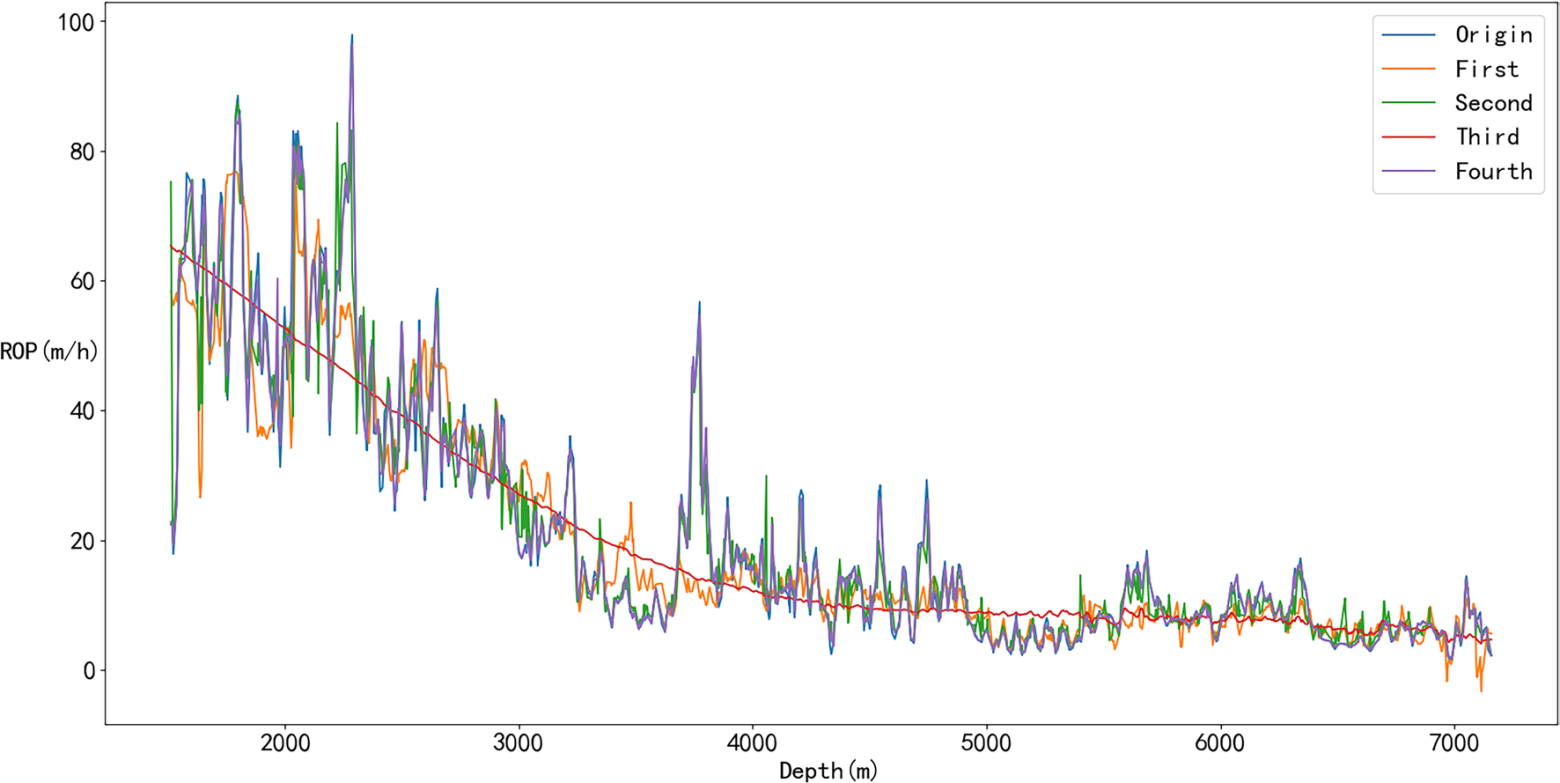
Prediction results of different hybrid models.

**Figure 26:**
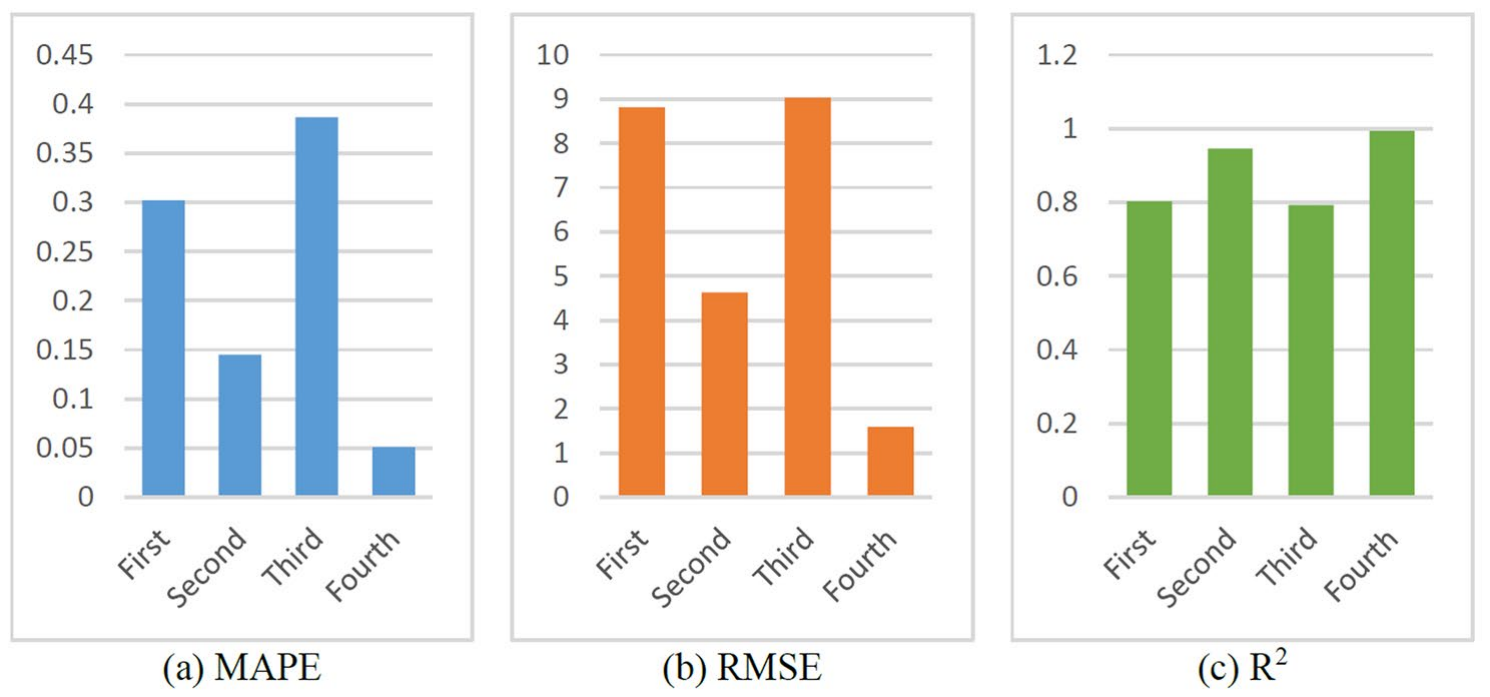
Evaluation indexes of different hybrid models.

The prediction results are shown in Figs. 25 and 26. As shown in Fig. 25, all four models achieved outstanding performances, thus verifying the effectiveness of the first hybrid model. The best ROP prediction model was selected by comparing the performance indices of all the models. The R^2^ values of the different models are close, but the Fourth model is slightly better than the other models. Moreover, the RMSE and MAPE values of the Fourth model are significantly lower than those of the other three models (Fig. 25 and Table 8), indicating that the prediction accuracies of the Fourth model are higher.

**Table 8:** Evaluation indexed of different hybrid models.

ROP model	MAPE	RMSE	R^2^
First	0.3021	8.8123	0.8026
Second	0.1449	4.6268	0.9456
Third	0.3865	9.0302	0.7927
Fourth	0.0509	1.5861	0.9936

Based on the above analyses, it can be concluded that the best hybrid model with high prediction accuracy is obtained only when the optimal physical and ML models are selected, which can provide important guidance for future ROP prediction modeling.

### Comparison of the effects of different dataset sizes on models

**Figure 27:**
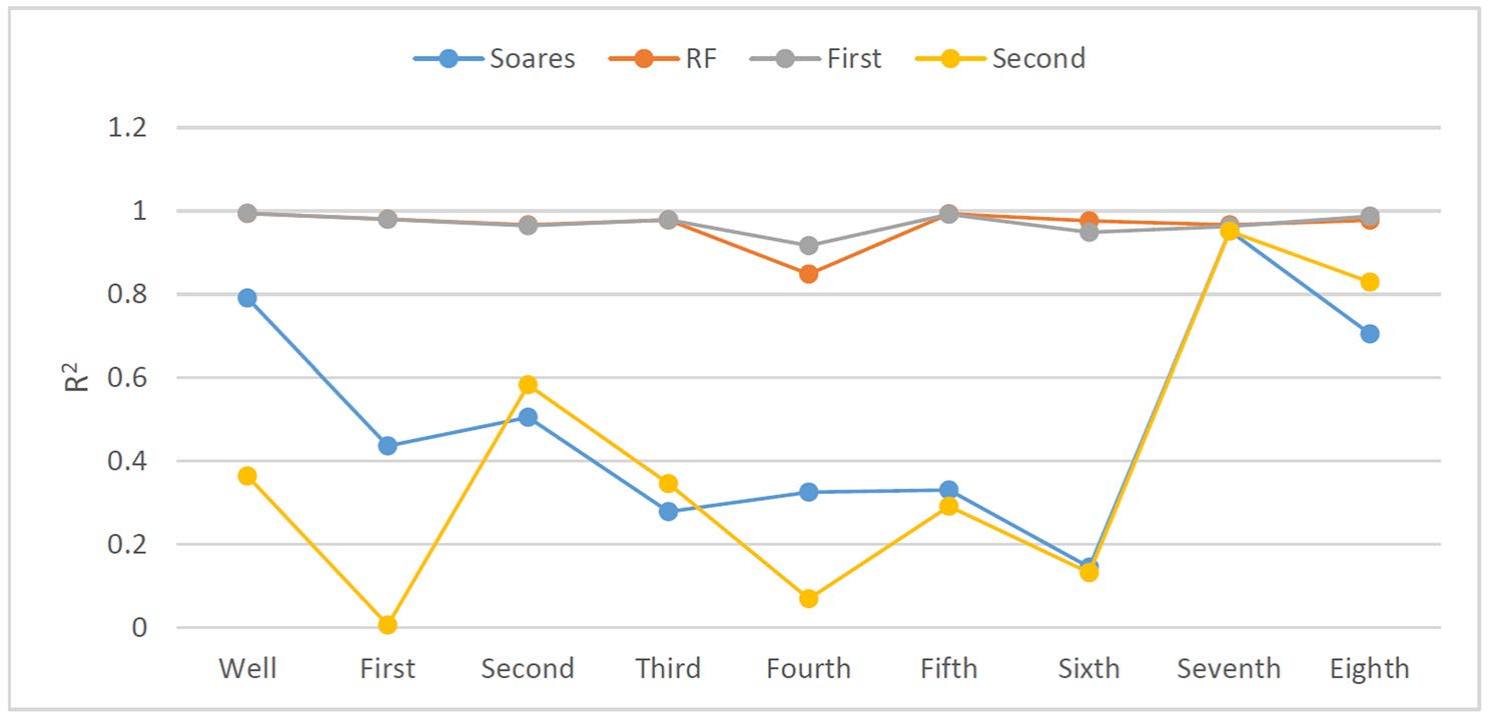
Prediction accuracy (R^2^) of different models with different dataset sizes.

In the drilling field, the ROP will change with rounding trip operations or when connections are made; consequently, the ROP prediction model may not reach enough accuracy when the dataset falls short of more than 5000 records in the above experiment. To study the effects of different dataset sizes on ROP models, an additional eight sets of tests were added to compare the full interval data according to the drilling depth of each bit (Table 3). The prediction results are shown in Figs. 26 and 27.

**Figure 28:**
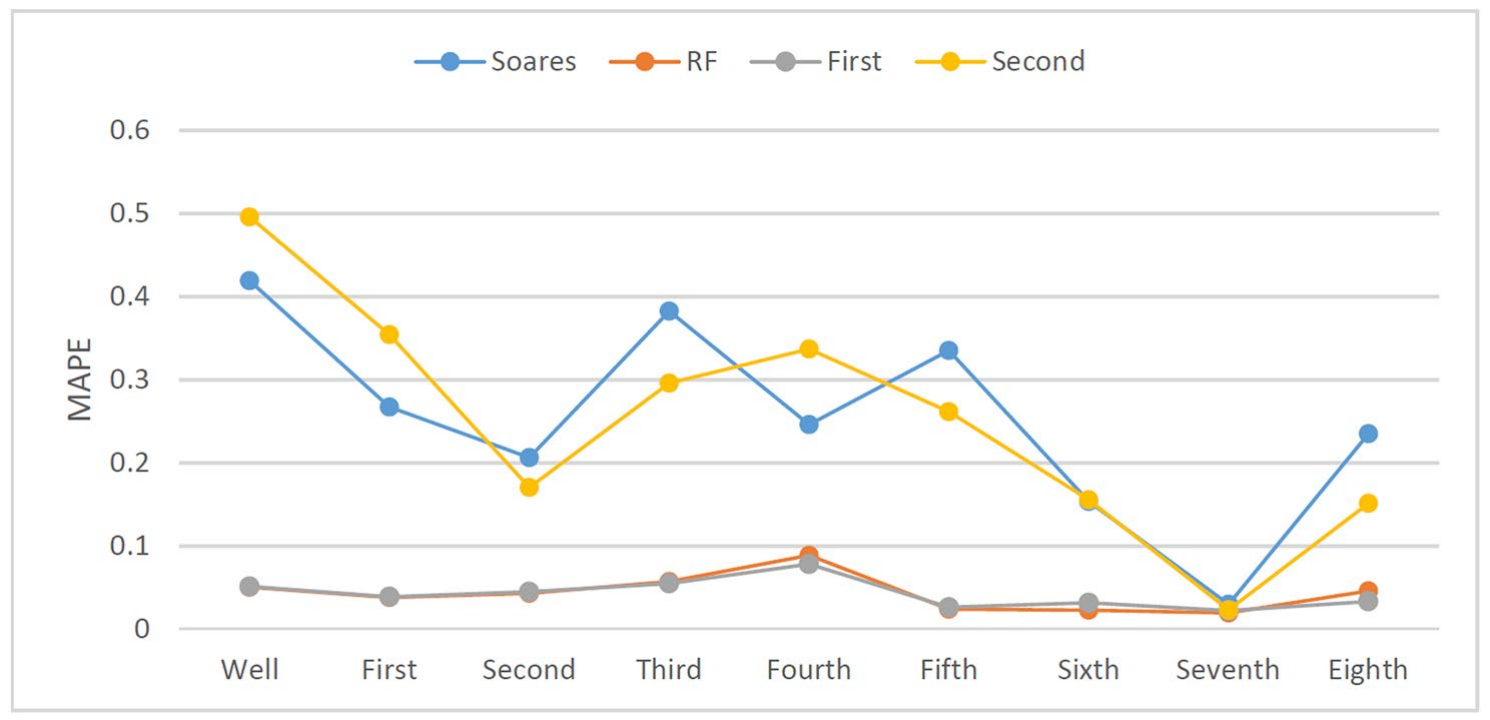
MAPE of different models with different dataset sizes.

**Figure 29:**
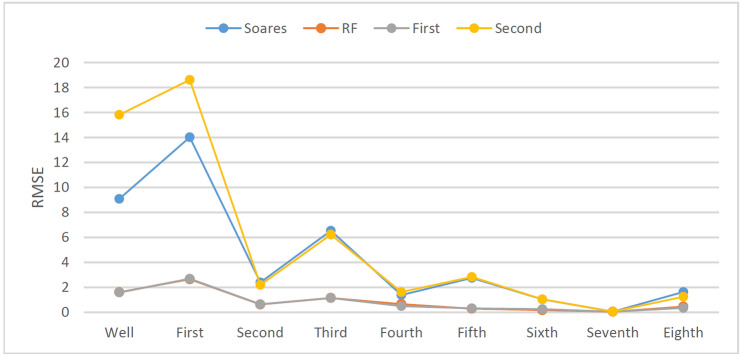
RMSE of different models with different dataset sizes.

As shown in Fig. 27, four different kinds of ROP models including the Soares model, the RF model, the First hybrid model and the Second hybrid model, were selected for comparison. According to Figs. 27, 28, and 29, the RF model and the First hybrid model, which are suitable for ROP prediction in the field, are less sensitive to changes in dataset size, but the accuracy of the Soares and the Second model is strongly affected by the dataset size. It is also proven that the First hybrid model has better generalization performance and accuracy and is more suitable for field application.

### Comparison of the effects of different data denoising methods on the model

**Figure 30:**
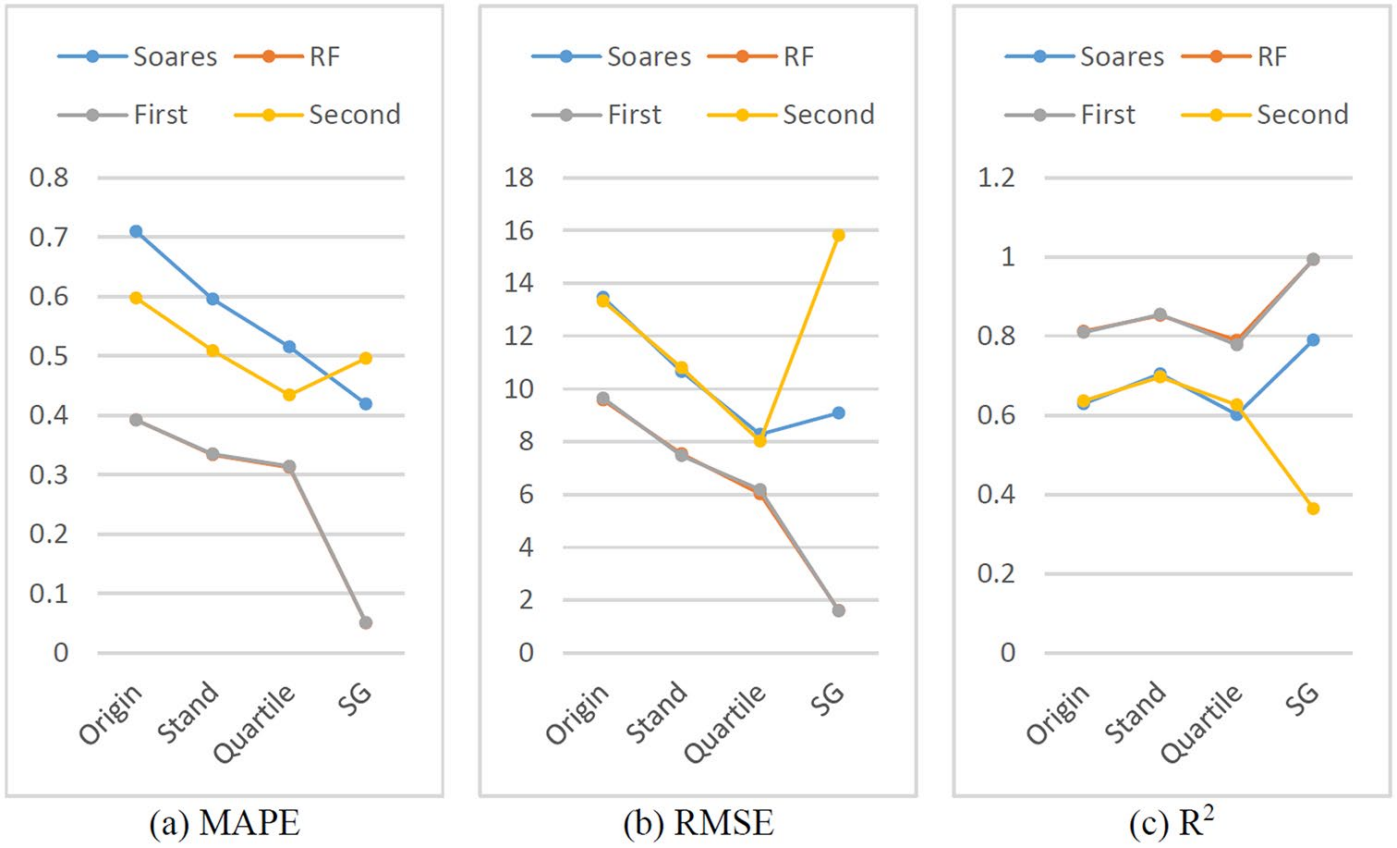
Evaluation indexes of Origin, Stand, Quartile, SG.

In the experiments in the above section, most of them used the whole well data after noise reduction by the SG algorithm to conduct the experiments. To compare the advantages and disadvantages of the SG algorithm and the effects of data denoising methods on the model, common standard deviation and quartile deviation were also used to conduct control experiments with the original data. The prediction results are shown in Fig. 30.

As shown in Fig. 30, from the overall trend, in the case of the same model prediction, the R2 is the highest, and the RMSE and MAPE are the lowest after SG noise reduction, except that the Second hybrid model is not suitable for the SG algorithm. In other words, the ROP prediction model should be retrained before it is used for new drilling areas, not only for physical models, ML models and hybrid models but also the data denoising methods and dataset sizes.

### Comparison of the effects of different data splitting methods on the model

**Figure 31:**
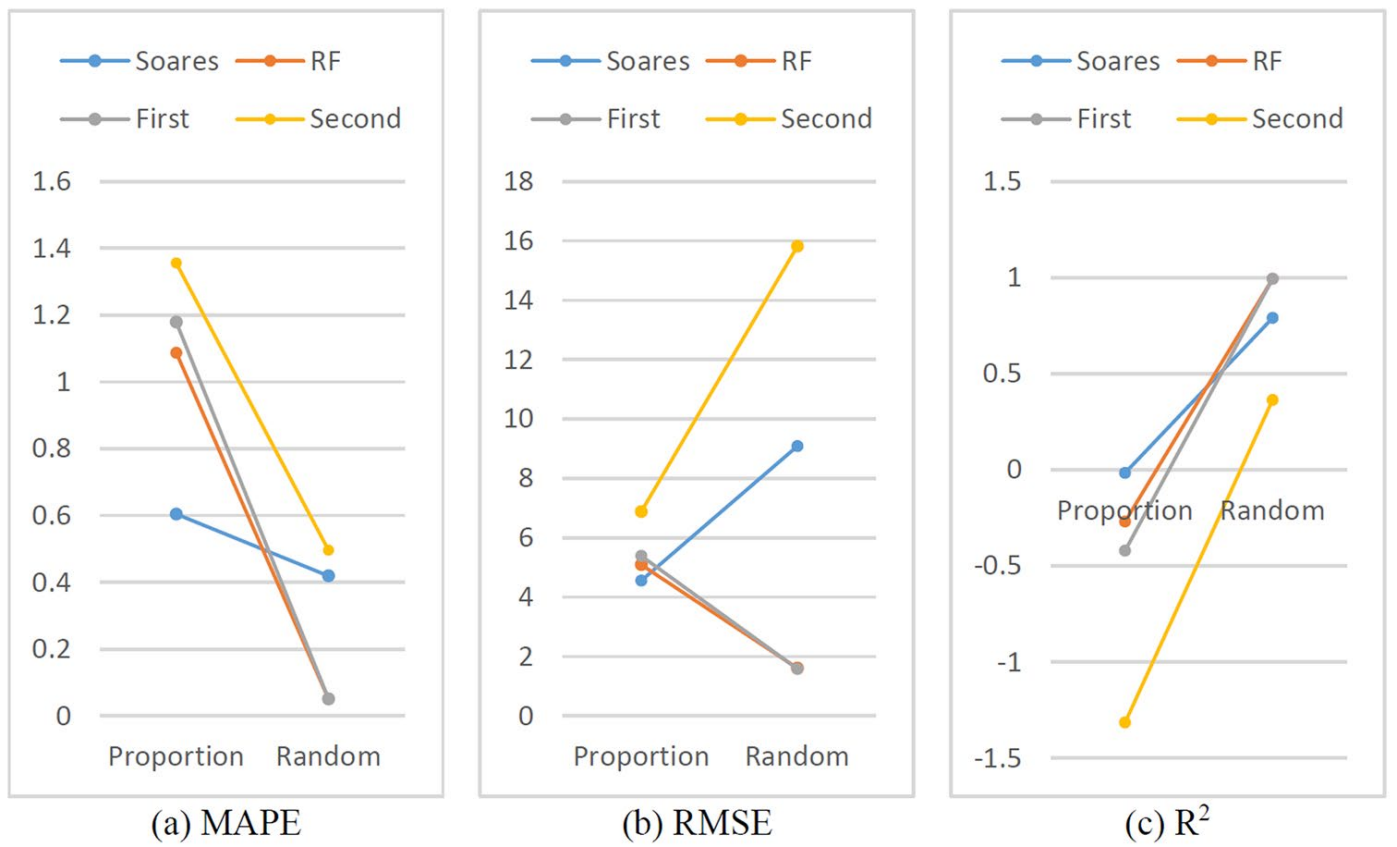
Evaluation indexes of proportion and random.

Generally, in the process of regression fitting or ML model training, randomly selected samples are used as training sets to ensure the representativeness of the samples and to ensure that all sample spaces can be included in the training set as much as possible to improve the accuracy and generalizability of the model. As shown in Fig. 31, both the training set and the test set were divided at 4:1, but one was divided according to the front-to-back ratio, and the other was divided randomly. All four models achieved higher accuracy in the case of random division than in the case of front-to-back ratio division.

In this study, a logging dataset was collected after real drilling, and the data of all the well segments were obtained. After the data noise reduction processing, the training set and the test set are randomly divided to obtain a better prediction effect. Moreover, in the real-time drilling process, the ROP prediction model can be trained only by drilled data, which include tens, hundreds or even thousands of meters of data that will be drilled in the next time. Perhaps this situation is similar to the division according to the front-to-back ratio, and cannot obtain a better drilling rate prediction effect like random division. How to ensure that the ROP prediction model can accurately predict the ROP of the adjacent drilling interval on the basis of the drilled interval data may be the focus of further research.

### Further discussion on hybrid models for ROP prediction

Judging from the above accuracy of the ROP predictions of the different models, the hybrid model has advantages in terms of prediction accuracy and interpretability, but it also has several limitations, such as the computational complexity of the hybrid models being greater than that of the single model. The computational complexity of hybrid models can be divided into p steps^57^, each corresponding to the training of a model (M_0_, M_1_, …, M_p_). The evaluated hybrid models are trained sequentially, and their computational complexity can be described as M_0_ + M_1_ + ··· + M_p_, where M_p_ is the computational complexity in ROP prediction. In this way, the four hybrid models are approximately two times more expensive than the single model, because the hybrid models use at least two kinds of models, including physical models and ML models. This work applied six combinations of hybrid models, Bingham, Eckel, Soares or ANN, SVM and RF models.

The three physical models were trained and fitted using the curve_fit function in scipy.optimize^58^, whose core method is non-linear least squares method to fit the ROM model. The computational complexity of it depends on the size and complexity problem, as well as the number of iterations required. Therefore, in this study, it is approximately assumed that the computational complexity is O(m^2^n), where n are the training examples and m is the total number of features/variables. The ANN training process has a complexity of O(emnk), where e is the number of epochs and k is the number of neurons^59^. The SVM training process has a complexity of O(nm), where n is the size of the dataset, and m represents the number of input features^60^. The RF training process has a complexity of O(Mmnlog(n)), where M is the number of trees, m is the number of features and n is the number of data samples in the training set^61^.

In the previous section, through ten-fold CV experiment, it was proven that the generalization of the model is not problematic in the Halahatang oil field. However, further in-depth research needs to be conducted in additional regions to further optimize the model and study generalizability.

## Conclusions

Through the above research and analysis, the following conclusions can be drawn:

1. The Soares model considering MD and Q was proven to be the most accurate for the Tarim Basin field among all physical models such as the Bingham and Eckel models. The R^2^ score of the Soares model is approximately 3 times greater than that of the Bingham model and 11.9% greater than that of the Eckel model.
2. Three ML algorithms were used to create machine learning models. Compared with these machine learning models, the RF model yielded the best results, with the highest correlation coefficient (R^2^ = 0.9934) and lowest prediction error (MAPE = 0.0501 and RMSE = 1.6072).
3. Four hybrid models, including residual modeling, integrate couple, simple average and bagging, were established. Most hybrid models showed higher accuracy than physical models and greater interpretability than conventional ML models.
4. The performance of the hybrid model with error compensation by ML was optimal among all the ROP prediction models. The R^2^ score improved greatly, specifically by approximately 25.77% in comparison with that of the Soares model. Moreover, the best hybrid model can be achieved when both physical and ML models with the best performances were selected for hybrid modeling.
5. The idea of a hybrid model with residual modeling and combining predictions from both physical and ML models are recommended for different drilling operations due to its clear physical meaning, awesome generalization capability, low modeling difficulty, and good interpretability. The RF algorithm is also recommended when using artificial intelligence because of its outstanding predictive accuracy.
6. The RF model and First hybrid model are relatively insensitive to dataset size compared with the other ROP models.
7. Most ROP models achieve better performance after denoising with the SG algorithm than after denoising with Stand deviation or Quartile deviation, a suitable denoising reduction method should be selected through experimentation and analysis.
8. Dividing the training set and test set according to the ration before and back, the accuracy of the trained model is far less than that of the random division model. To employ the ROP model in real-time drilling, how to make the ROP prediction model accurately predict the ROP of the adjacent drilling interval on the basis of the drilled interval data may be the focus of further research.
9. Further studies can focus on the optimizing drilling parameters to increase the ROP. In addition, in this study, the selected ML algorithms were mostly common ML methods, that can be combined with physical models by deep learning, transfer learning, reinforcement learning, large model and other algorithms.

## Data Availability

The utilized data in this study is available upon reasonable request from the corresponding author.

## Abbreviations

ROP: Rate of penetration
ML: Machine learning
Bagging: Bootstrap aggregating
WOB: Weight on bit
RPM: Revolutions per minute
ANN: Artificial neural network
SVM: Support vector machine
RF: Random forests
ELM: Extreme learning machines
DL: Deep learning
MLP: Multilayer perceptron
BP: Back-propagation
IGA: Improved genetic algorithm
DC: Dc exponent
DCN: Normal dc exponent
${g}_{p}$: Formation pressure gradient
${G}_{f}$: Formation fracture pressure gradient
$$\mu $$: Poisson’s ratio
Q: Drill fluid flow rate
DT: Drilling time
HL: Hook load
SPP: Stand pipe pressure
MD: Measured depth
TVD: True vertical depth
${d}_{b}$: Drill-bit diameter
${d}_{f}$: Drill-bit footage
ECD: Drilling fluid equivalent density
SG: Savitzky–Golay
PCC: Pearson correlation coefficient
MIC: Maximal information coefficient
Bingham: Bingham’s model
Eckel: Eckel’s simplified model
Soares: Cesar Soares’s model
SVR: Support vector regression
CART: Categorical regression trees
RMSE: Root mean square error
MAPE: Mean absolute percentage error
R^2^: Coefficient of determination
TRF: Trust region reflective algorithm
